# Heterogeneity in effect size estimates

**DOI:** 10.1073/pnas.2403490121

**Published:** 2024-07-30

**Authors:** Felix Holzmeister, Magnus Johannesson, Robert Böhm, Anna Dreber, Jürgen Huber, Michael Kirchler

**Affiliations:** ^a^Department of Economics, University of Innsbruck, A-6020 Innsbruck, Austria; ^b^Department of Economics, Stockholm School of Economics, SE-113 83 Stockholm, Sweden; ^c^Department of Occupational, Economic, and Social Psychology, University of Vienna, A-1010 Vienna, Austria; ^d^Department of Psychology and Center for Social Data Science, University of Copenhagen, DK-1353 Copenhagen, Denmark; ^e^Department of Banking and Finance, University of Innsbruck, A-6020 Innsbruck, Austria

**Keywords:** heterogeneity, generalizability, metascience

## Abstract

A typical empirical study involves choosing a sample, a research design, and an analysis path. Variation in such choices across studies leads to heterogeneity in results that introduce an additional layer of uncertainty, limiting the generalizability of published scientific findings. We provide a framework for studying heterogeneity in the social sciences and divide heterogeneity into population, design, and analytical heterogeneity. Our framework suggests that after accounting for heterogeneity, the probability that the tested hypothesis is true for the average population, design, and analysis path can be much lower than implied by nominal error rates of statistically significant individual studies. We estimate each type's heterogeneity from 70 multilab replication studies, 11 prospective meta-analyses of studies employing different experimental designs, and 5 multianalyst studies. In our data, population heterogeneity tends to be relatively small, whereas design and analytical heterogeneity are large. Our results should, however, be interpreted cautiously due to the limited number of studies and the large uncertainty in the heterogeneity estimates. We discuss several ways to parse and account for heterogeneity in the context of different methodologies.

## Significance

In conducting empirical research in the social sciences, the results of testing the same hypothesis can vary depending on the population sampled, the study design, and the analysis. Such variation, referred to as heterogeneity, limits the generalizability of published scientific findings. We estimate heterogeneity based on 86 published meta-scientific studies. In our data, the estimated design and analytical heterogeneity are substantive and of at least the same magnitude as sampling uncertainty, whereas population heterogeneity is smaller. The results suggest low generalizability of the empirical findings under consideration across different study designs and statistical analyses.

Designing an empirical study, collecting or sourcing data, and analyzing data call for making decisions in heaps, many of which are up to the researcher’s discretion (1). This flexibility, dubbed researcher degrees of freedom, opens the door to a “garden of forking paths” (2) involving many branches (choices) and countless designations (empirical results). Yet, empirical research methods in the social sciences typically involve relying on one particular sample, choosing one out of many possible study designs, and reporting the results for one of many possible analysis pipelines. In light of a “publish or perish” culture in academia (3–5), scholars have a strong incentive to exploit researcher degrees of freedom to obtain statistically significant results and selectively report empirical estimates that maximize the publication potential (6–10). It is now acknowledged that the opportunistic misuse of researcher degrees of freedom—commonly referred to as selective reporting and *p*-hacking—implicates increased false-positive rates (11–13) and inflated effect sizes (14–16). Alongside publication bias (17–19), low statistical power (20–23), and HARKing (24, 25), *p*-hacking has been argued to be one of the “four horsemen of the reproducibility apocalypse” (26). Several large-scale direct replication projects (27–29) suggest that on average, replication effect sizes are only about 50% of the published effect sizes in empirical research in psychology and economics. Scientific reforms such as open, transparent, and confirmatory research practices have been advocated—and implemented to a greater or lesser extent—to reduce systematic bias in the published literature and “rein in the four horsemen” (30–34).

Even if researchers and journals adopt a culture of confirmatory research practices (35, 36) to remedy systematic bias in the scientific knowledge accumulation, the scholarly community faces another obstacle on its way toward reliable empirical evidence: the doubt about the generalizability and robustness of reported results to alternative populations, research designs, and analytical decisions (37–42). Typically, empirical studies only capture tiny snapshots of the range of possible results, and common estimates of the uncertainty about these snapshots do not account for the uncertainty due to the flexibility in choosing a sample, a research design, and an analysis path during a research project. The magnitude of this unaccounted-for uncertainty—commonly referred to as heterogeneity—depends on how much results vary across populations, alternative research designs, and alternative analysis paths.

This paper provides a framework for defining and estimating the various sources of heterogeneity in the empirical social sciences, categorizing heterogeneity into three distinct types: population, design, and analytical heterogeneity. While some recent studies estimate population heterogeneity based on multilab replication studies (43, 44), there is a lack of work systematically estimating design and analytical heterogeneity across studies. We review the evidence on the three types of heterogeneity based on research settings where each type is isolated and systematic bias in effect sizes (due to *p*-hacking and publication bias) has been ruled out by design. In our data, analytical and design heterogeneity are substantial and larger than population heterogeneity. Notwithstanding, our results should be interpreted cautiously due to the limited number of included studies and the uncertainty of the estimates. We discuss the implications of our findings and different ways of coping with heterogeneity in scientific practice.

## Framework

While the term heterogeneity may be used with slightly different meanings across various contexts, we adhere to the definition that is specific to the random-effects meta-analytic model, where a distinction is made between the within-study variance (*σ*^2^; i.e., the sampling error) and the between-study variance (*τ*^2^; heterogeneity) (45). In this realm, heterogeneity is uniformly defined as the variation in effect size estimates over and above sampling variation, i.e., observing study outcomes being more different from one another than would be expected due to chance alone. The square root of the between-study variance (*τ*) has the intuitive interpretation of the SD of the distribution of true effect sizes across the studies included in the meta-analysis. As our framework rests on the conceptualization of heterogeneity in a random-effects meta-analysis, it rests on the assumptions of the random-effects model (45).

Heterogeneity can be quantified in terms of *τ* and expressed in both absolute and relative terms. While the absolute magnitude of heterogeneity is important, it is difficult to compare estimates across studies utilizing different effect size measures. Estimates of *τ* can only be reasonably compared across meta-analyses if they utilize the same standardized effect size measure. As the effect size measurement varies across the empirical studies reviewed below, we focus on quantifying heterogeneity in relative terms to facilitate comparability, but we also report estimates in absolute terms. A common way to quantify heterogeneity in relative terms is to express the overall variability in effect sizes, i.e., *τ*^2^ + *σ*^2^, in within-study variance (*σ*^2^) units. This ratio is commonly denoted as *H*^2^ and can be conceived of as a variance inflation factor due to heterogeneity. In what follows, we favor the square root-transformed version of *H*^2^ to facilitate interpretability (i.e., to characterize heterogeneity in SD units rather than in variance units) and refer to it as the heterogeneity factor (H), which is defined as

$$H=\sqrt{\frac{\sigma^{2}+\tau^{2}}{\sigma^{2}}.}$$

This expression has previously been proposed as a heterogeneity measure in the context of random-effects meta-analyses (46, 47) and relates to the commonly reported heterogeneity measure *I*^2^, defined as the percentage of the total variability in effect size estimates attributable to heterogeneity, i.e.,

$$I^{2}=\frac{\tau^{2}}{\sigma^{2}+\tau^{2}}\;\;\to \;\;H=\sqrt{\frac{1}{1-I²}}.$$

The commonly referenced cutoff values of 25%, 50%, and 75% for *I*^2^ are used to indicate small, medium, and large heterogeneity (48, 49) and translate into cutoff values for *H* of 1.15, 1.41, and 2.00, respectively. *H* is the factor that the sampling SE needs to be multiplied by to account for heterogeneity; *H* = 1 implies homogeneity, and, e.g., *H* = 2 implies that accounting for the uncertainty due to between-study variation will double the sample SE of an individual study.

Heterogeneity in effect sizes may stem from various sources: Study outcomes might be heterogeneous across samples drawn from different populations (population heterogeneity), estimates can vary depending on the study design used to address a particular hypothesis (design heterogeneity), and effect sizes may differ depending on the analysis path implemented (analytical heterogeneity). For studies that rely on prospective data collections, such as experiments, the three types of heterogeneity relate to degrees of freedom in different stages of the research process. These include deciding i) which population(s) to use to draw a sample from, ii) which research design to implement, and iii) how to analyze the sampled data. Design heterogeneity includes variation in what List (50) refers to as the “experimental environment,” such as heterogeneity due to experimenter effects and the degree of anonymity. For empirical studies that rely on observational data, it may not be clear where to draw the line between design and analytic decisions. For ease of exposition, we consider all researcher decisions made after choosing which raw data to use as part of the analytical domain in empirical studies.

Each type of heterogeneity can be isolated and quantified by implementing adequate research designs. By allowing for variation in only one dimension (e.g., the study designs) while holding the other dimensions (e.g., the population and the analysis) constant, the magnitude of heterogeneity due to the different sources of variation can be examined without being conflated with other sources of variability. Our empirical review reports estimates of heterogeneity expressed in terms of the heterogeneity factor (*H*) separately for population, design, and analytical heterogeneity (based on meta-analyses isolating either of the three sources by design). Assuming that the heterogeneity estimates due to different sources of variability are linearly additive in variances, the overall heterogeneity (in absolute terms) is given by *τ*^2^ = *τ_P_*^2^ + *τ_D_*^2^ + *τ*_A_^2^, where *τ_s_* denotes the between-study variance due to variability across populations (*s* = *P*), designs (*s* = *D*), or analysis paths (*s* = *A*), respectively. Importantly, the way *H* is constructed remains unchanged irrespective of the source of variability, i.e., whether *H* pertains to one of the three sources of heterogeneity or any combination thereof is solely governed by the research design. All empirical analyses reported below determine the heterogeneity factor *H* based on the within-study variance (*σ*^2^) estimated as part of the meta-analytic random-effects model.

Consider an individual study, study *j*, testing a hypothesis based on a single population, a single research design, and a single analysis path. Two types of inference are feasible: i) the study can provide inference about the tested hypothesis being true for the population, design, and analysis in question, such that the variance of the effect size estimate is equal to *σ_j_*^2^; or ii) the study can provide inference about the tested hypothesis being true for the average population, design, and analysis path that could be employed to test the underlying hypothesis. We refer to the latter as “meta-inference” about the “meta-hypothesis” and the average effect size as the “meta-effect.” If study *j* seeks to provide meta-inference about the phenomenon under consideration, the total variance of study *j*’s estimate of the meta-effect, *ν_j_*^2^, is given by *ν_j_*^2^ = *σ_j_*^2^ + *τ_P_*^2^ + *τ_D_*^2^ + *τ*_A_^2^, which incorporates the uncertainty due to heterogeneity into the hypothesis test. While it is not obvious per se which of the two inference types should be preferred, a strong argument can be made for incorporating the variance due to design and analytical heterogeneity into statistical inference. Despite the common practice of basing inference on a single design and a single analysis pipeline, we argue that there is no apparent reason why researchers should be more interested in whether a hypothesis is true for a specific analysis and study design rather than whether it is true for the universe of equally justifiable designs and analyses. For population heterogeneity, however, the question of whether to prioritize meta-inference depends on the population of interest to the researcher and which population the sample included in the study is drawn from. If a random sample of participants from the population of interest is included in the study, a relevant variance of the meta-effect would be given by *ν_j_*^2^ = *σ_j_*^2^ + *τ_D_*^2^ + *τ*_A_^2^, which would incorporate the variance due to design and analytical heterogeneity but not the variance due to population heterogeneity.

**Fig. 1.**
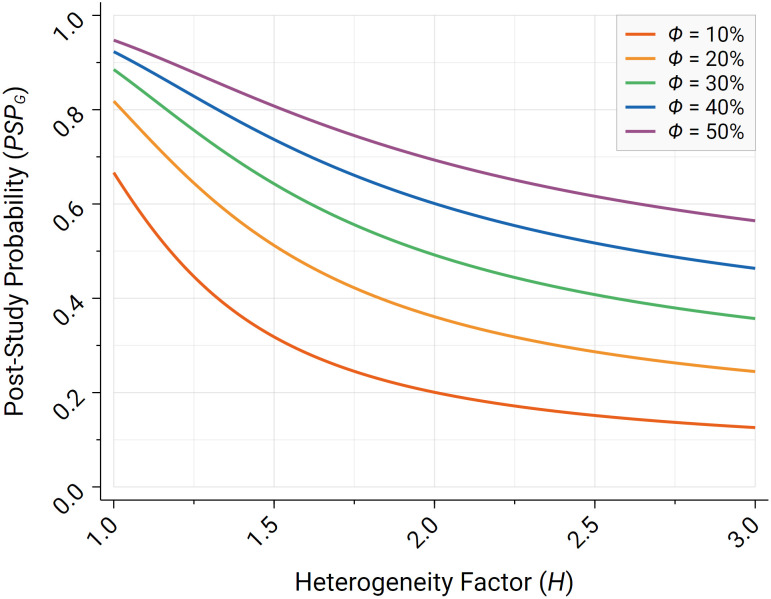
The figure illustrates the effective *PSP_G_*, i.e., the ratio of true positive results to the total number of positive classifications in the presence of heterogeneity, for different prior probabilities for the alternative hypothesis being genuinely true (*ϕ*), as a function of the heterogeneity factor *H* for a two-tailed *z*-test with nominal statistical power of *π* = 90% at the nominal *α* = 5% level.

Following Maniadis et al. (51, 52) and Butera et al. (53), we define the poststudy probability (*PSP*) as the fraction of statistically significant associations that are genuinely true, i.e., the probability of the tested hypothesis being true after observing a statistically significant effect. The *PSP* can be defined as i) the *PSP* of the hypothesis being true for the specific population, research design, and analysis path considered in the test or as ii) the *PSP* of the meta-hypothesis being true. The latter, which we refer to as *PSP_G_*, can be conceived as a measure of the generalizability of an individual study. In Fig. 1, we illustrate how *PSP_G_* is impacted by the heterogeneity factor *H* for different priors of a tested hypothesis being true (*ϕ*) (see the *Materials and Methods* section for further details). *PSP_G_* decreases strongly with *H*; e.g., for a prior of *ϕ* = 30%, the probability of the meta-hypothesis being true, for an individual study with 90% (nominal) statistical power to detect the hypothesized effect size at the 5% level, goes from 88.5% in the case of homogeneity (*H* = 1) to 49.2% for *H* = 2. For lower priors, the impact of heterogeneity on *PSP_G_* is even stronger. Thus, in the presence of high heterogeneity, the generalizability of statistically significant results in individual studies is inevitably low.

### Empirical Estimates of Population, Design, and Analytical Heterogeneity.

**Fig. 2.**
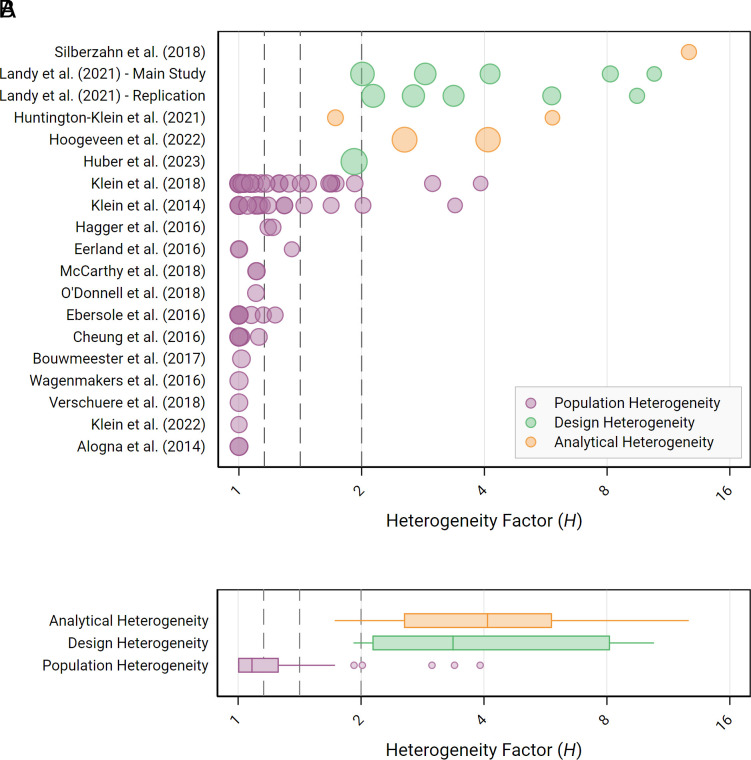
Empirical estimates of population, design, and analytical heterogeneity. (*A*) The figure shows estimates of the heterogeneity factor *H* for 70 estimates from 13 papers isolating population heterogeneity (54–66), 11 estimates from two papers isolating design heterogeneity (67, 68), and five estimates from three papers isolating analytical heterogeneity (69–71). The vertical reference lines indicate benchmark levels for small, medium, and large heterogeneity based on *I^2^* values of 25% (*H* = 1.15), 50% (*H* = 1.41), and 75% (*H* = 2), respectively. (*B*) The figure shows box plots of the distribution of heterogeneity factors *H*, separated by the source of heterogeneity, illustrated in panel (*A*).

To gauge the extent of heterogeneity in empirical social science research, we review heterogeneity estimates—reestimated using random-effects meta-analysis—of published crowd science projects. We outline the inclusion criteria and the estimation procedures in the *Materials and Methods* section; details about the individual studies are provided in *SI Appendix*, sections 1–3. The results are illustrated in Fig. 2, which also indicates benchmarks for low (*I*^2^ = 25%, *H* = 1.15), medium (*I*^2^ = 50%, *H* = 1.41), and high heterogeneity (*I*^2^ = 75%, *H* = 2.00) as commonly used in meta-analysis to classify the magnitude of heterogeneity (48, 49). Estimates of the heterogeneity measures *τ*, *I*^2^, and *H* (together with their corresponding 95% CIs) and the results of Cochran’s *Q*-test for each meta-analysis reviewed in our empirical analysis are tabulated in *SI Appendix*, Table S1.

### Population Heterogeneity.

Population heterogeneity can be measured by implementing the same research design and analysis in independent samples drawn from different populations and estimating the SD in true effect sizes across samples (*τ*) in a random-effects meta-analysis. This is what has been pioneered in the *ManyLabs* (ML) replication studies and various Registered Replication Reports (RRRs) in psychology, which are ideal for measuring population heterogeneity. Our analysis involves four ML studies (54–57) and nine RRRs (58–66). As some of the included studies report results for multiple effects, our sample of studies isolating population heterogeneity comprises 70 meta-analyses.

The estimated population heterogeneity varies substantially across the meta-analyses in our sample, with a large number of estimates (19/70 = 27.1%) indicating homogeneity (*H* = 1.00) and some estimates unveiling substantial heterogeneity of up to *H* = 3.91 (with 4/70 estimates exceeding the threshold value of *H* = 2.00, indicative of large heterogeneity). The median *H* across the 70 meta-analyses is 1.08; a large fraction of the estimates are in the small to moderate heterogeneity range. Cochran’s *Q*-test rejects the null hypothesis of homogeneity at the 5% level for 21 (30%) of the sampled meta-analyses and at the 0.5% level for 14 (20%) of the sampled meta-analyses (42). Some meta-analyses (46/70 in 4/13 papers) are based on effect sizes measured in terms of Cohen’s *d*; heterogeneity can be reasonably compared across studies in absolute terms (*τ*) for this subsample. The estimated *τ* varies between 0.00 and 0.69 for these estimates, with a median of 0.06. Note that the distributions of *H* and *τ* estimates of population heterogeneity are subject to some upward bias due to following the convention to truncate *τ^2^* estimates at zero (i.e., to prevent the identification of excess homogeneity) (46, 47). For genuinely homogeneous effect sizes, randomness would lead to both negative and positive estimates of *τ^2^.* This upward bias can be substantial whenever the fraction of meta-analyses with zero estimated heterogeneity is large—as is the case for our sample of studies isolating population heterogeneity.

### Design Heterogeneity.

Design heterogeneity can be measured by randomly allocating experimental participants sampled from the same population to different research designs while holding the analysis constant and estimating the SD in true effect sizes across research designs (*τ*) in a random-effects meta-analysis. We identified two studies that implemented such a research design, reporting the results of 11 meta-analytic estimates for six empirical claims: Landy et al. (67) tested five hypotheses on moral judgments, negotiations, and implicit cognition in 12 to 13 experimental designs each (once in a “main study” and once in a replication). Huber et al. (68) examined the effect of competition on moral behavior across 45 crowd-sourced experimental protocols. The estimates of *H* for the 11 meta-analyses reported in the sampled studies (Fig. 2) suggest that the extent of design heterogeneity is substantial. The estimates of *H* vary between 1.92 and 10.44, with a median of 3.36, and Cochran’s *Q*-test rejects the null hypothesis of homogeneity (*P* < 0.005) for each of the 11 meta-analyses. In our data, design heterogeneity is substantially larger than population heterogeneity. Design heterogeneity of this magnitude adds substantial uncertainty to estimates based on an individual design and implies low generalizability across study designs. All estimates of design heterogeneity are in Cohen’s *d* units: The estimated *τ* varies between 0.14 and 0.78, with a median of 0.23.

### Analytical Heterogeneity.

An effective means to estimate analytical heterogeneity involves randomly allocating independent analysts to test the same hypothesis on mutually exclusive random subsamples of a dataset and estimate the SD in true effect sizes across analysts (*τ*) in a random-effects meta-analysis. To the best of our knowledge, no studies have employed this method yet. Studies closest to this ideal are those relying on the multianalyst approach, where different analysts independently test the same hypothesis on the same data. Our review involves three papers that fulfill our inclusion criteria (see *Materials and Methods* for details), examining the variability in effect sizes due to analytical flexibility for five hypotheses (69–71).

As the analysts in multianalyst studies are required to estimate the effect in question using the same data, the analysts’ individual estimates are not independent. Despite the violation of the model assumptions, we use random-effects meta-analyses to estimate *τ* and *H* as an approximation of the analytical heterogeneity for the sampled multianalyst studies. A recent multianalyst study in biology (72) also used this method to estimate the heterogeneity of results across analysts. The estimates reported in Fig. 2 should be interpreted cautiously since relying on the random-effects meta-analytic model will underestimate heterogeneity for correlated observations (as the within-study variation will be lower in the case of dependent observations). The estimated analytical heterogeneity is large in our data, with *H* estimates ranging from 1.72 to 12.69, with a median of 4.08. Cochran’s *Q*-test is statistically significant (*P* < 0.005) for each of the five meta-analyses. In the *Materials and Methods* section, we provide a robustness test on the estimates of analytical heterogeneity, showing that the high estimate of 12.69 for Silberzahn et al. (70) is driven by two outliers in terms of sampling variance; removing these outliers reduces the estimated heterogeneity factor *H* to 2.72. In the sampled studies, analytical heterogeneity is substantial, in the same ballpark as the estimates for design heterogeneity, and substantially larger than our estimate of population heterogeneity. None of the estimates of analytical heterogeneity are in standardized effect size units, and the analytical heterogeneity estimates cannot be reasonably compared in absolute terms across the studies.

### Limitations.

Our empirical estimations come with several limitations and should be interpreted with due care. First, it is worth emphasizing that the estimated heterogeneity factors *H* carry significant uncertainty for all three types of heterogeneity: Heterogeneity estimates can be highly sensitive to outliers in effect sizes and sample variances of the individual studies included in the meta-analyses [as illustrated above for the study by Silberzahn et al. (70)]. Therefore, the individual estimates of *H* in Fig. 1 should be interpreted with great caution. Second, all studies included in our review are from psychology, except for two studies (68, 69) from economics. It may well be possible that heterogeneity varies across fields in the social sciences. Third, multianalyst studies have been criticized for overestimating the analytical variation, e.g., due to ambiguity about the studied research question (73, 74). Our estimates of heterogeneity can thus be considered to be subject to heterogeneity themselves, implying that the conclusions drawn about the extent of generalizability of empirical claims are not necessarily generalizable beyond the set of studies included in our systematic review. Fourth, the heterogeneity estimates are derived from a small sample of studies, especially those for design and analytical heterogeneity, and the number of studies differs markedly across the three types of heterogeneity. Fifth, comparisons across types of heterogeneity may be considered unjust given that the range of observed variation might be a function of the limited set of studies used for estimating each type of heterogeneity; population heterogeneity might, for instance, be small just because the sampled populations in the included studies are relatively similar.

Notwithstanding, it is important to note that even the smallest estimates obtained from our review of the literature point toward considerable heterogeneity due to the variability in designs (*H* = 1.92) and analyses (*H* = 1.72). It is also worth emphasizing that a typical empirical study will involve heterogeneity due to all three sources investigated above, which implies that the overall level of heterogeneity may be even higher than the estimates reported in Fig. 2.

## Coping with Heterogeneity in Scientific Practice

Our framework and empirical results illustrate how heterogeneity can lower generalizability and why it is important to parse and cope with heterogeneity in scientific practice (37–40). Different research methodologies entail a distinct set of promises and pitfalls as to how heterogeneity can be accounted for in inference about the meta-hypothesis. In this section, we discuss the opportunities and challenges faced and provide guidance on how to cope with heterogeneity due to different sources in the context of different methodologies: i) the traditional “one population–one design–one analysis” approach, ii) multianalyst studies, iii) multiverse-style analysis, iv) standard meta-analysis, and v) a methodological advancement that we refer to as preregistered prospective meta-analysis.

### One Population–One Design–One Analysis Approach.

The estimated levels of design and analytical heterogeneity in our data indicate that the generalizability of individual studies can be low. An important first step to overcome the adverse effects of unwarranted generalizations is to increase awareness of the importance of heterogeneity to pave the way toward more nuanced assessments and interpretations of empirical results (75). Considerations of heterogeneity and generalizability should also find their way into editorial guidelines to inform both authors and reviewers about their importance. Methodological standardization, sharper theoretical predictions, and clearer alignment of theoretical conceptualizations and empirical instrumentalizations can also narrow down the set of plausible research designs and analytical choices, thereby reducing heterogeneity (37–39).

Another possibility would be to directly incorporate the added uncertainty due to heterogeneity into inference. Directly incorporating heterogeneity would imply multiplying the estimated SE by a sensible estimate of *H*. A conservative interpretation of the estimates based on the studies in our sample suggests that accounting for heterogeneity would approximately double sample SE and CI. However, it seems hasty to recommend a specific multiplier due to the limited number of included studies, the uncertainty surrounding our *H* estimates, and the limited information about whether and how *H* varies across methods and fields. Further evidence is needed. A promising method of obtaining further evidence of analytical heterogeneity and its magnitude (*H*) is the multianalyst approach.

### Multianalyst Studies.

Multianalyst studies offer a methodology to map and gauge analytical heterogeneity (76–78) and were used as a basis for estimating *H* due to analytical heterogeneity in our empirical review. While the multianalyst approach brings with it the potential to cope with analytical heterogeneity by pooling results based on various justifiable analysis paths, we are not aware of any multianalyst study proposing or conducting joint inference. For the multianalyst approach to become an appropriate methodology that allows going beyond demonstrating the variability of effect sizes and conclusions, it should be refined and steered in the direction of pooled hypothesis tests. The challenge in devising joint inference procedures across the analysis paths implemented by the analysts is that the estimates are not independent, causing difficulties in estimating both the uncertainty of the mean effect size and heterogeneity across analysts. Both simple pooled tests and more sophisticated tests should be explored. A simple pooled hypothesis test would rely on the average *t-*/*z*-value of all analyses. While the mean *t*-/*z*-statistic will not incorporate analytical heterogeneity, it can be a straightforward and sensible means to pooled inference whenever the number of analysts is sufficiently large (as the impact of heterogeneity becomes negligible due to the fact that the analytical variation gets divided by the square root of the number of analysts). A more sophisticated test would rely on bootstrapping (79) both the analysis paths and the data sample to estimate the SEM effect size to construct a *z*-test statistic for a pooled hypothesis test. Such a bootstrap test would incorporate both the sampling variance and analytical heterogeneity while taking into account the correlation in effect size estimates across analysts.

As emphasized in the discussion of our estimates of analytical heterogeneity, the question of how to handle “outlier analysis paths” plays a crucial role in estimating meta-effects and heterogeneity in multianalyst settings. It is essential to have methods and procedures in place to ensure quality control of the analysis paths chosen by analysts and to determine whether or not these paths qualify as legitimate to address the hypothesis in question (80). We recommend that multianalyst studies report estimates of the extent of analytical heterogeneity in terms of *H* (or *I*^2^) obtained via a random-effects meta-analysis and in terms of the ratio between the SD of effect size estimates across analysts and the mean SE as introduced by Huntington-Klein et al. (63). The latter measure, which can be converted into a proxy measure of *H* (see the *Materials and Methods* section), is used in a robustness test reported below. A viable alternative approach to estimating analytical heterogeneity is multiverse-style analysis.

### Multiverse Analysis.

A practical alternative to the multianalyst approach is multiverse-style analyses (81–83), in which a multitude of justifiable analytical choices are factorially combined to map the analytical space. Multiverse-style analyses can be conceived of as modeling the theoretical variability of estimates, whereas multianalyst studies gauge analytic variability “in the wild.” A multianalyst study can be thought of as a weighted multiverse analysis with weights determined based on the frequency with which analysts implement (combinations of) particular analytical choices. While multiverse-style analyses are on the rise in psychology and related fields (84), we are not aware of any studies reporting indicators of analytical heterogeneity such as *H*. We recommend routinely reporting heterogeneity estimates in the context of multiverse-style analyses. The same indicators of heterogeneity as proposed for multianalyst studies apply.

Multiverse-style analyses typically plot the distribution of effect sizes or *P*-values across analysis paths and provide descriptive statistics as the focal outcomes, but there is also a limited set of literature on how to aggregate individual results into pooled hypothesis tests. In introducing the multiverse approach to psychology, Steegen et al. (82) mention the possibility of using the average *P*-value across analysis paths as a pooled hypothesis test. However, averaging *p*-values is inexpedient whenever effect size estimates point in different directions, as is often the case in multianalyst and multiverse-style studies. Using the average *t-*/*z*-value instead corresponds to the simple test mentioned above. Simonsohn et al. (83) propose approaching the joint inference problem using permutation and bootstrapping techniques and put forward a number of pooled tests based on median effect sizes and the fraction of significant results. While the median estimate is more robust to outliers, the mean effect size could be argued to be a more relevant pooled measure if all implemented analysis paths are equally legitimate. There is scope for more work on appropriate and practical joint inference methods, with multianalyst studies and multiverse-style analyses facing similar challenges.

Multiverse-style analyses offer a viable tool for estimating and incorporating analytical heterogeneity into statistical inference. Yet, similar to the multianalyst approach, the expediency of multiverse-style methods hinges on the identification of the analytical space (84); reliable mechanisms for doing so are needed. Moving toward preregistered multiverse analysis would also be an important step to avoid analytic forks and choice points being chosen selectively.

### Standard Meta-Analysis.

The standard tool for incorporating heterogeneity into inference is applying random-effects meta-analysis, pooling effect size estimates across different (independent) studies. Typically, standard meta-analyses synthesize estimates based on varying populations, study designs, and analyses such that various sources of heterogeneity are modeled jointly. Pooling many studies in a random-effects meta-analysis has the important advantage that the uncertainty due to heterogeneity is substantially reduced, and with a sufficiently large number of studies, precise estimates of meta-analytic effect sizes can be obtained. However, as currently practiced, standard meta-analyses have important limitations. The estimated results are affected by publication bias and *p*-hacking (16–19, 85), the results are sensitive to the decision of which studies to include/exclude, and the scope for intentionally or unintentionally *p*-hacking meta-analytic results is large. With the rise of preregistration, detailed preanalysis plans, and Registered Reports, the validity of standard meta-analysis may increase over time. In particular, meta-analyses that only include results from Registered Reports have the potential (conditional on proper inclusion criteria) to provide unbiased meta-analytic effect sizes, which incorporate the uncertainty due to heterogeneity from various sources. Below, we discuss the opportunities and challenges involved in transitioning toward preregistered prospective meta-analysis to further enhance the validity of meta-analytic estimates.

### Preregistered Prospective Meta-Analysis.

An alternative to conducting traditional meta-analyses based on the existing literature would be heading toward fewer but much larger empirical studies in which reasonable research designs, analysis paths, and relevant populations are systematically varied as part of an encompassing research design. We think of such studies as prospective meta-analyses to distinguish them from traditional ones. When analyzing such studies using random-effects meta-analytic models, heterogeneity is incorporated into the SE of the meta-analytic effect size. The systematic variation of populations, designs, and analytical choices can be used to identify sources of heterogeneity, yielding important insights to build more complete and nuanced theories (39). Importantly, such studies should be preregistered to limit researcher degrees of freedom and can involve elements of crowd science to reach the necessary scale (86, 87).

The *ManyLabs* studies (54–57) in psychology can be thought of as examples of pioneering work in this direction, although primarily concerned with studying population heterogeneity. Another example is Huber et al. (61), systematically varying experimental designs across random samples from the same population. Prospective meta-analyses could also be augmented by the multianalyst approach to facilitate the incorporation of analytic heterogeneity. If independent research teams, for instance, decide on both their research design and a preferred analysis pipeline, and the design and analysis proposals of all teams are implemented in independent random subsamples, the meta-analytic mean will account for both analytical and design heterogeneity. Likewise, analytical heterogeneity could be integrated by implementing multiverse-style analyses for the various research designs considered in a prospective meta-analysis by estimating the meta-analytic effect across analysis paths for each design and subsequently aggregating the estimates in a random-effects model. The meta-analytic estimate would also incorporate population heterogeneity if the various design and analysis proposals were implemented in different populations.

We advocate moving toward conducting more research in the form of preregistered prospective meta-analyses. Conceptually, our plea for more large-scale studies systematically integrating heterogeneity is similar in spirit to a recent proposal by List (86)—referred to as “option C,” as a mnemonic for an approach beyond A/B testing—, recommending the use of field experiments to generate evidence for policymaking before implementing policies on a larger scale. List (88) argues for complementing the typical initial small efficacy test under ideal conditions with a test under natural conditions, which directly produces the evidence needed to evaluate the policy after scaling. Integrating option C thinking into research practice implies larger and more costly data collections in exchange for more efficient and comprehensive knowledge generation and avoids scaling up initially promising projects that will not thrive on a grand scale. Shifting evidence generation toward relying more on preregistered prospective meta-analyses would involve similar trade-offs: fewer but more costly studies in exchange for expedited and more comprehensive evidence generation—a trade-off that seems worthwhile. However, steering common research practices in the direction of prospective meta-analysis calls for adapting the “rules” of the academic enterprise: Funding bodies would need to prioritize large-scale data collections, the academic reward system (including, e.g., hiring decisions) would need to adapt to acknowledge crowd science contributions, and the infrastructure for conducting team science would need to be strengthened.

## Discussion

Aside from the literature reviewed as part of our empirical exercise, a wealth of meta-analytic studies in psychology (and related fields) report heterogeneity estimates as part of random-effects meta-analyses. For the sake of comparison, the results of two studies are worthwhile to mention: van Erp et al. (89) sourced more than 700 meta-analyses published in the Psychological Bulletin and reported a median *I*^2^ estimate of 71%; Stanley et al. (90) reviewed a convenience sample of 200 meta-analyses published in the same journal and reported a median *I*^2^ of 74%. These *I*^2^ estimates—pooling all potential sources of heterogeneity—translate into heterogeneity factors (*H*) of 1.86 and 1.96, respectively. However, these estimates are difficult to draw on for our purpose, and the comparability with our estimates is limited since heterogeneity estimates in meta-analyses based on the published literature will be impacted by publication bias and *p*-hacking (16–19, 85). In our review of results, we only draw on studies that are, by design, free from publication bias and obvious incentives for *p*-hacking. This literature is still at an early stage, and our results should be interpreted with due care; yet, drawing some preliminary conclusions appears tenable. In our data, population heterogeneity is comparatively small, which is consistent with two recent studies estimating population heterogeneity based on multilab replication studies (43, 44). However, both design and analytical heterogeneity are large in the studies included in our review.

A typical empirical study will be associated with all three sources of heterogeneity, implying even higher levels of uncertainty not captured by SE. However, we would be reluctant to simply add up our three estimates for different sources of heterogeneity as they are based on different types of studies. The estimates of population and design heterogeneity are based on experimental studies, whereas the estimates of analytical heterogeneity are based on observational data research. We would expect less analytical heterogeneity for the typical experiment than for the typical observational study due to fewer analytical choice points encountered on average (8, 9). Conversely, it is more difficult for observational data studies to cleanly separate the research design from analytical decisions; analytical heterogeneity may incorporate (part of) the variability of “design elements,” whereas the remaining design heterogeneity may be lower than for experiments. More research is needed to gauge the relative importance of various types of heterogeneity in different methodologies. The tentative insights gained from our review suggest that total heterogeneity in psychology and economics research can be expected to be substantial.

The sizeable analytical heterogeneity observed in our review also implies a wide scope for selective reporting of favorable results. While *p*-hacking is often thought of as marginally affecting results around the significance thresholds (7–9), the extent of observed analytical heterogeneity suggests that there is potential for much larger systematic bias in published effect sizes. Similarly, design heterogeneity implies that researchers may be able to selectively report results for research designs that deliver the desired results. “Design hacking” could manifest itself in opportunistically choosing the experimental design that can be expected to maximize the chances of finding statistically significant results based on, e.g., piloting different protocols and parameterizations. Thus, all pilot studies and related tests that have been used to inform the eventual research design should be explicitly reported to counteract design hacking; ideally, studies should be preregistered before conducting any pilot tests such that the piloting choices are explicitly incorporated into the overall research design.

For our estimates of population heterogeneity, the reviewed multilab replication studies feature a relatively high share of null results, which may limit the scope of heterogeneity. Another important caveat is that these studies are typically based on university student samples from different Western countries, which may involve lower population heterogeneity than in other settings (91–93). Put differently, our comparatively low estimates of population heterogeneity might be subject to population heterogeneity itself. To what extent one should incorporate population heterogeneity into the reported uncertainty of individual studies also depends on the population to which the researcher wants to generalize the results (94, 95). When conducting an experiment on university students, it seems fair to expect that results are generalizable to similar student populations. However, it may not be justifiable to generalize the findings to other populations, such as students in different countries or the general population. To avoid overgeneralization, empirical investigations should ideally start with representative samples of the population for which the results ought to be informative, in which case the population heterogeneity will be “absorbed” by the sampling SE of the study. For population heterogeneity, it may also be important to study whether and to what extent effect sizes systematically vary across populations rather than generalizing results beyond the population studied in a specific study. Gauging the variability in effect sizes across populations is of direct interest, can inform future research agendas, and may be policy-relevant.

The common one population–one design–one analysis approach has dominated social sciences for a long time. In this approach, scientific progress is made in small steps (41). The publication of one study might inspire follow-up studies, which in turn trigger follow-up studies, etc. With scientific evidence pertaining to a narrowly defined set of hypotheses being published sequentially, reaching a broader perspective on heterogeneity and generalizability could take years. The process of sequential publication further involves the threat that flawed initial results could steer an entire subdiscipline in the wrong direction, which, in turn, could lead to research waste and impede efficient knowledge accumulation (96, 97). This risk increases with heterogeneity, and estimates of design and analytical heterogeneity in our study suggest that the generalizability of a typical individual study in psychology and economics is low. A complement to the traditional knowledge generation process would be relying more on large-scale prospective meta-analyses, involving systematic data collection for different research designs and implementing various legitimate analysis paths. While adopting such a methodology at scale would involve more resource-intense research agendas and call for a culture change in the scientific enterprise, it may bring with it the potential to speed up scientific progress and bypass some of the drawbacks of the traditional model.

## Materials and Methods

### Included Studies.

#### Population heterogeneity.

We reviewed the literature for *ManyLabs* (ML) replication studies and RRRs in psychology, which are ideal for measuring population heterogeneity, and included all ML and RRRs using random-effects meta-analysis and with available data on effect sizes and SE for each included lab. We included ML1-4 (54–57) and nine RRRs published in *Perspectives on Psychological Science* and *Advances in Methods and Practices in Psychological Science* (58–66). We did not include ML5 (98) due to a lack of data availability. As ML1–3 and several RRRs report results for multiple effects, our sample comprises 70 separate meta-analyses for which we estimated population heterogeneity. See *SI Appendix*, section 1, for details of the included studies.

#### Design heterogeneity.

To the best of our knowledge, there are only two studies (67, 68) that vary the experimental design to test the same hypothesis in random subsamples to isolate design heterogeneity in a random-effects meta-analysis. Both studies, reporting results on six different hypotheses, are included in our analysis of design heterogeneity. The five hypotheses examined in the study by Landy et al. (67) were tested in a main study and an independent replication study each, implying that the number of estimates on design heterogeneity from that study is 10, and the total number of estimates is 11). See *SI Appendix*, section 2, for more details of the included studies.

#### Analytical heterogeneity.

We reviewed all multianalyst studies in the social sciences for which data on effect sizes and SE are available for each analyst, and effect sizes are measured in the same units across analysts. We found three papers that meet our criteria: Silberzahn et al. (70), Huntington-Klein et al. (69), and Hoogeveen et al. (71). In total, these papers examine analytical variability for five different hypotheses. We identified five more published multianalyst studies, but these did not meet our inclusion criteria: the study by Bastiaansen et al. (99) details the variation in analytic decisions across analysis teams but does not report estimates pertaining to each of the proposed analysis pipelines; the study by Botvinik-Nezer et al. (100) was excluded as the primary outcome reported by analysis teams is a binary classification of whether the hypotheses are supported by the data, but no effect size measure is reported; the study by Schweinsberg et al. (101) was excluded since the individual results by analysts are not available in standardized effect-size units but only in terms of *z*-scores; the study by Menkveld et al. (102) was excluded as the data are yet embargoed; and the study by Breznau et al. (103) was excluded as the research teams reported various results for the same hypothesis, and it is not clear which effect size estimate to include for each team. Note that the reported variation in results across analysts is also very large for the five excluded studies. See *SI Appendix*, section 3, for more details of the included studies.

### Estimation of Results for Included Studies.

We reestimated the random-effects meta-analytic models for each included study based on the original data. In *SI Appendix*, Table S1, we provide detailed results for each included meta-analysis, comprising the *Q*-test, whether effect sizes were measured in Cohen’s *d* units, the between-study variation (*τ* and its 95% CI), the within-study variation (*σ*), the ratio between the between- and within-study variation (*τ*/*σ*; which we refer to as the heterogeneity ratio *HR*), *I*^2^ and its 95% CI, and *H* and its 95% CI. If not indicated otherwise in *SI Appendix*, sections 1–3, we were able to precisely (computationally) reproduce the results reported in the papers. As such, our study provides—as a “side product”—evidence on the computational reproducibility (104–108) of large-scale meta-scientific results.

To keep things simple and easily replicable, we created copies of the relevant input data for the meta-analyses (i.e., the effect size estimate and the corresponding SE for each study included in the meta-analysis) for each paper based on the original data (all of which are publicly available under a CC-by license). These copies of the original data constitute our raw data. All data and analysis scripts used to generate the results reported in the main text and *SI Appendix* are available at our project’s OSF repository: osf.io/yegsx.

Random effects meta-analyses were estimated using the metafor package (v-4.4.0) (109) in R (v-4.3.2) (110). Estimates for the CI around the heterogeneity measures *τ*^2^, *I*^2^, and *H*^2^ were based on the *Q*-profile method (111) implemented using the confint() function shipped with the metafor package. For all papers reporting the results of meta-analyses (i.e., all papers on population or design heterogeneity), we used the same estimator for *τ*^2^ as used in the original paper. Most of these papers relied on the restricted maximum likelihood estimator (112); but one study used the DerSimonian–Laird (113) estimator, and one used the Hartung–Knapp (114) estimator.

For multianalyst studies, heterogeneity estimates were based on the restricted maximum likelihood estimator. Note that estimating a random-effects model on multianalyst-style data is unconventional, as discussed in the main text. Hence it does not come as a surprise that none of the multianalyst studies included in our review reported the results of a random-effects meta-analysis; however, a recent multianalyst study in biology (72) used a meta-analytic random-effects model to estimate the heterogeneity of results across analysts. The estimated heterogeneity measures *τ*^2^, *I*^2^, and *H*^2^ for multianalyst studies can be interpreted as lower bound estimates as they are derived based on the within-study variance that would be observed if the effect size estimates reported by multiple analysts were independent observations; if the sampling variances of the multiple analysts are correlated (which is the case for multianalyst studies, since analysts based their estimates on the same dataset), the actual within-study variance is lower, and the between-study variance is higher.

### Robustness Tests on Analytical Heterogeneity.

Huntington-Klein et al. (69) introduced the ratio between the SD of effect size estimates across analysts and the mean SE as a measure of the analytical heterogeneity in multianalyst studies. This measure can be interpreted as a proxy for the ratio of the between-study variation and the within-study variation (*HR*) and can be converted to a proxy measure of *H* by taking the square root of 1 plus the squared ratio; to distinguish the two measures from *HR* and *H* obtained from the estimates of random-effects meta-analyses, we denote them as *HR_P_* and *H_P_*. In *SI Appendix*, Table S2, we report both *HR_P_* and *H_P_* for the multianalyst studies included in our review. *HR_P_* varies between 1.48 and 3.98 for the multianalyst studies, with a median of 3.07; *H_P_* varies between 1.79 and 4.11 for the multianalyst studies, with a median of 3.23. Note that while the between-study variation (*τ*) estimated in the random-effects meta-analysis is a lower bound of the SD in effect sizes across analysts, the proxy *H_P_* may exceed *H* as estimated using a random-effects meta-analysis as the estimated within-study variation (*σ*) in the random-effects meta-analysis can differ from the average SE of estimates generated by the analysts. The proxy *H_P_* is quite similar to *H* based on the random-effects meta-analysis for four of the five multianalyst estimates but differs substantially for Silberzahn et al. (70). This is due to two outliers in terms of low SE strongly affecting the estimated within-study variance in the random-effects meta-analysis (as the individual effects are weighted by the inverse of their variance). This is an indication that the estimate of *H* for Silberzahn et al. (70) should be interpreted very cautiously; but also the proxy *H_P_* indicates substantial analytical heterogeneity. Removing the two outliers for Silberzahn et al. (70) (implying *k* = 27 effect size estimates) results in the following heterogeneity estimates in a random-effect meta-analysis: *Q*(26) = 130.3, *P* < 0.001; *τ* = 0.107, *I*^2^ = 86.5%, *H* = 2.717, *HR* = 2.527; the proxy measures based on ref. 69 remain qualitatively unchanged (*H_P_* = 1.721, *HR_P_* = 1.401).

### Estimating the PSP in Light of Heterogeneity.

Consider a generic two-tailed *z*-test with power *π* to detect an effect *θ* at a type-I error rate α. The effect size *θ* (measured in *z*-score units in the generic test) corresponds to the noncentrality parameter *δ* = |*z*_*α*/2_| + |*z*_*β*_|, where *z_p_* denotes the *p*th quantile of the inverse cumulative standard normal distribution, and *β* = 1 − *π* denotes the false negative rate. Assuming that the true effects to be estimated are homogeneous, *θ*_i_ ~ *N*(*μ*_0_, σ^2^) under the null hypothesis H_0_ (with *μ*_0_ indicating the test value and *σ*^2^ denoting the test's sampling variance); under the alternative hypothesis H_A_, *θ*_i_ ~ *N*(*δ*, σ^2^).

Now suppose there is variation in the true effect size above and beyond the uncertainty that is accounted for by the test's sampling variance (σ_i_^2^). Put differently, effect size estimates are subject to an additional source of uncertainty—heterogeneity—such that the overall variance of study *i*’s estimate *θ*_i_ is given by *ν*_i_^2^ = σ^2^ + *τ*^2^. The heterogeneity estimate *τ*^2^ indicates the variance of the genuine effect, such that *θ*_i_ ~ *N*(*μ*_0_, *ν*_i_^2^) under H_0_ and *θ*_i_ ~ *N*(*δ*, *ν*_i_^2^) under H_A_.

Instead of quantifying the extent of heterogeneity in absolute terms (i.e., in terms of *τ*^2^ or *τ*, respectively), it is expedient to denote it relative to the test’s sampling variance (σ_i_^2^). Following the notational conventions applicable to random-effects meta-analysis (45), we define a heterogeneity factor *H* as

$$H=\sqrt{\frac{\sigma^{2}+\tau^{2}}{\sigma^{2}},}$$

which is equivalent to the square root of *H*^2^, a commonly used measure of heterogeneity reported in meta-analyses. *H*^2^ can be interpreted as a “variance inflation factor” due to heterogeneity (46, 47), i.e., as the factor a test’s sampling error σ_i_ needs to be multiplied by to incorporate the uncertainty due to heterogeneity into statistical inference. We prefer *H* over *H*^2^ as thinking about heterogeneity in terms of SD units appears more convenient than thinking about it in terms of variance units. *H*^2^ is defined as the relative excess of the *Q*-statistic over its degrees of freedom, i.e., *H*^2^ = *Q*/(*k* − 1). Following conventions, we presume *H* = max(1, *H*) but acknowledge that this prevents identifying excessive homogeneity—i.e., less variability than would be expected due to chance (46).

We distinguish between nominal and effective error rates below, where the effective error rates are the observed error rates after accounting for heterogeneity. The effective false positive rate α’ in a two-tailed *z*-test in the presence of heterogeneity (expressed in terms of the heterogeneity factor *H*) is given by

$$\alpha^{\prime }=2\cdot \Phi \left(\frac{z_{\alpha /2}}{H}\right),$$

where Φ(·) indicates the cumulative standard normal distribution, and *z*_α/2_ denotes the α/2-percentile of the inverse cumulative standard normal density function Φ^−1^(·) (i.e., the critical value of a two-tailed *z*-test at a nominal significance threshold α). It follows that α’ > α for any *H* > 1. Correspondingly, the effective false negative rate *β*′ is given by

$$\beta^{\prime }=\Phi \left(\frac{z_{\beta }}{H}\right).$$

The *PSP* of the hypothesis being true is defined as the ratio of true positive results to the total number of positive classifications, which implies that the *PSP* is a function of the prior probability *ϕ* for the alternative hypothesis being genuinely true, i.e.,

$$PSP=\frac{\phi \cdot \left(1-\beta \right)}{\left(1-\phi \right)\cdot \alpha +\phi \cdot \left(1-\beta \right)}\;.$$

Note that *PSP* is equivalent to the inverse of the false discovery rate, i.e., our results could just as well be expressed in terms of the rate of false positive classifications.

Since heterogeneity inflates the effective type-I error rate (for any nominal α-level in a two-tailed test) and the effective type-II error rate (for any nominal *β* > 0.5), it follows that the *effective* poststudy probability, *PSP_G_*, is given by

$$PSP=\frac{\phi \cdot \left(1-\Phi \left(\frac{z_{\beta }}{H}\right)\right)}{\left(1-\phi \right)\cdot 2\cdot \Phi \left(\frac{Z_{\frac{\alpha }{2}}}{H}\right)+\phi \cdot \left(1-\Phi \left(\frac{z_{\beta }}{H}\right)\right)}.$$

Since the cumulative normal density Φ(·) is convex in the domain (−∞, 0], it follows that *PSP_G_* < *PSP* for any *H* > 1. Fig. 1 illustrates *PSP_G_* as a function of *H* for various levels of the prior *ϕ*. *PSP_G_* can be conceived as an indicator of the generalizability of an individual study, measuring the probability of the meta-hypothesis being true, conditional on an individual study reporting a statistically significant result.

## Supplementary Material

Appendix 01 (PDF)

## Data Availability

The data used to estimate population, design, and analytical heterogeneity and the analysis scripts generating all results, figures, and tables reported in the main text and *SI Appendix* are available at the project’s OSF repository (https://osf.io/yegsx/) (115).
