# Supplementary material for: Heterogeneity in effect size estimates
Source: Proc Natl Acad Sci U S A. 2024 Jul 30;121(32):e2403490121. doi: 10.1073/pnas.2403490121 (PMC11317577; doi:10.1073/pnas.2403490121)
Supplement: Supplementary file 1 — Appendix 01 (PDF) [file pnas.2403490121.sapp.pdf]

PNAS

Supporting Information for

Heterogeneity in effect size estimates

Felix Holzmeister[1,*], Magnus Johannesson[2], Robert Böhm[3,4], Anna Dreber[1,2], Jürgen Huber[5], Michael Kirchler[5]

[1] Department of Economics, University of Innsbruck, Innsbruck, Austria. [2] Department of Economics, Stockholm School of Economics, Stockholm, Sweden. [3] Faculty of Psychology, University of Vienna, Vienna, Austria. [4] Department of Psychology and Center for Social Data Science, University of Copenhagen, Copenhagen, Denmark [5] Department of Banking and Finance, University of Innsbruck, Innsbruck, Austria.

* Correspondence should be addressed to:

Felix Holzmeister
University of Innsbruck, Department of Economics
Universitätsstraße 15, 6020 Innsbruck Austria
felix.holzmeister@uibk.ac.at

This PDF file includes:

SI Methods
References
Tables S1–S2

SI Methods

Below, we provide further details about the estimations of population heterogeneity (Section 1), design heterogeneity (Section 2), and analytical heterogeneity (Section 3) for each included study.

1 Population Heterogeneity

To consolidate evidence on population heterogeneity, we consider the results of 70 random-effects meta-analyses (based on multi-site data collections using the same study protocols) from two different sources: *(i)* registered replication reports (published in either *Perspectives on Psychological Science* or *Advances in Methods and Practices in Psychological Science*) and *(ii)* the *ManyLabs* projects. As to the sample of registered replication reports, our sample comprises 18 meta-analyses from nine multi-site studies[1–9]. Another RRR by Colling et al.[10] is not included in our analyses as the meta-analytic results are based on multilevel multivariate meta-analytic methods (rather than random-effects meta-analyses). The sample of *ManyLabs* studies comprises 52 meta-analytic estimates published in *ManyLabs1* through *ManyLabs4*[11–14]; *ManyLabs5*[15] is not included in our analyses due to a lack of data availability.

1.1 Registered Replication Reports

Alogna et al. (2014)[1]. The paper reports the results of 31 (RRR1) and 22 (RRR2) independent direct replications of Study 4 and Study 1 in Schooler & Engstler-Schooler[16], respectively. For both meta-analyses, the paper reports Cochran's Q-test, τ, I^2, and H^2, but no confidence intervals around τ, I^2, and H^2. The original data is unavailable in the project's OSF repository (osf.io/ybeur). We generated the raw data files for our reanalysis of both RRRs based on the information provided in Figures 2 and 3 in the paper (i.e., the effect size and 95% confidence interval per laboratory). We determined the standard error per lab by dividing the difference between the effect size estimate and both the lower and upper bound of the 95% CI by the critical value of the normal distribution first and then averaging the two standard error estimates (pertaining to the lower and upper bound, respectively). As the estimates and 95% CIs in the figures are provided with only two digits after the comma, our re-estimates are subject to some degree of imprecision due to rounding; yet, the discrepancies between the heterogeneity estimates reported in the paper and our re-estimates turn out to be negligible for the heterogeneity measures.

Bouwmeester et al. (2017)[2]. This registered replication report involves 21 independent direct replications of Study 7 in Rand, Greene, & Novak[17]. We only consider the paper's primary analysis, i.e., the analysis without any exclusion criteria (intention-to-treat analysis). The paper reports Cochran's Q-test and I^2 but no confidence interval around I^2. All original data and analysis scripts are available at osf.io/9tpgx. While the heterogeneity estimates and the meta-analytic effect size are computationally reproducible, the 95% CI around the meta-analytic effect size differs slightly between Bouwmeester et al.'s report and our re-estimation.

Cheung et al. (2016)[3]. This registered replication report involves 16 independent direct replications of Study 1 in Finkel et al.[18] The paper reports meta-analyses on five dependent variables, all of which are included in our reanalysis. The paper reports Cochran's Q, τ, I^2, and H^2 for each meta-analysis, but no confidence intervals around τ, I^2, and H^2. Heterogeneity estimates are based on the Hartung-Knapp[19] estimator. All original data and analysis scripts are available at osf.io/3nz7j. Re-estimating the heterogeneity measures based on the original data results in slightly different estimates as compared to what is reported in the original paper for all dependent measures except for "subjective commitment" (manipulation check); yet, the discrepancies are negligible (less than two percentage points for I^2, and less than 0.02 units for H^2).

Eerland et al. (2016)[4]. The paper reports meta-analytic results based on 12 independent direct replications of Study 3 in Hart & Albarracín[20]. The paper reports meta-analyses on three dependent variables, all of which are included in our analysis. For each meta-analysis, the paper reports Cochran's Q-test, τ, I^2, and H^2, but no confidence intervals around τ, I^2, and H^2. All original data and analysis scripts are available at osf.io/hx7a4. All estimates reported in the original paper are computationally reproducible.

Hagger et al. (2016)[5]. The paper reports a multi-lab (k = 23) preregistered replication of the ego-depletion paradigm reported in Sripada, Kessler, & Jonides[21]. The paper includes meta-analyses on various outcome measures; we only include what appears to be the main analyses of the paper: the meta-analyses on differences in reaction times (RT) and reaction time variability (RTV) for the full samples, but not the meta-analyses on self-reported outcomes. The paper reports Cochran's Q-test and I^2, but no confidence intervals around I^2. All original data and analysis scripts are available at osf.io/kh85v. While the raw data available in the original study's OSF repository appears to be consistent with what is shown in Figures 1 and 2 in the paper (except for the mapping of lab names and results, which seems to be mixed up in the forest plots in the paper), the heterogeneity estimates reported in the paper cannot be (computationally) reproduced: the re-estimated Q-statistic is slightly smaller for both analysis, involving that I^2 in both re-estimations is about five percentage points smaller than what is reported in the paper.

McCarthy et al. (2018)[6]. This registered replication report reports the result of 22 independent direct replications of Experiment 1 in Srull & Wyer[22]. We only consider the paper's two primary analyses, i.e., *(i)* judgments of Ronald's hostility and *(ii)* judgments of ambiguously hostile behaviors. The paper reports Cochran's Q, τ, and I^2, but no confidence intervals around τ and I^2. The original data and code are available at osf.io/mcvt7. All estimates reported in the original paper are computationally reproducible.

O'Donnell et al. (2018)[7]. The paper reports the results of meta-analyses involving 23 independent direct replications on a variant of Experiment 4 in Dijksterhuis & van Knippenberg[23]. We only consider the paper's primary analysis. However, we do not include meta-analyses on the moderation effect of gender (which is also referred to as a primary analysis in the paper). The paper

reports Q, τ, I^2, and H^2 estimates, but no confidence intervals around τ, I^2, and H^2. All original data and analysis scripts are available at osf.io/fyptm. All estimates reported in the original paper are computationally reproducible.

Verschure et al. (2018)[8]. This registered replication report involves 19 independent direct replications of Experiment 1 from Mazar, Amir, and Ariely[24]. We only consider the paper's primary analysis. The paper reports estimates for Cochran's Q, τ^2, and I^2, but no confidence intervals around τ^2 and I^2. The original data and code are available at osf.io/mcvt7. All estimates reported in the original paper are computationally reproducible.

Wagenmakers et al. (2016)[9]. This registered replication report comprises 17 independent direct replications of Study 1 from Strack, Martin, & Stepper[25]. The original paper does not report any statistics on heterogeneity but focuses on the meta-analytic effect size estimate based on a random-effect meta-analysis only. All original data and analysis scripts are available at osf.io/h2f98. All estimates reported in the original paper are computationally reproducible.

1.2 Many-Labs Studies

Ebersole et al. (2016)[11]. *ManyLabs3* comprises multi-site direct and conceptual replications of 10 effects involving data collections across 20–21 independent sites each. In our review, we only consider the 10 primary effects[26–35] examined in *ManyLabs 3*, but not the three added interaction effects on elaboration likelihood[28], credentials and prejudice[31], and self-esteem and subjective distance[32]. The paper reports Cochran's Q-test and I^2 for each studied effect, but no confidence intervals around I^2. The original data and analysis scripts are available at osf.io/ct89g. For all but three studies[33–35], the original scripts actually implement meta-analysis based on correlation coefficients rather than standardized mean differences. For these studies, we base our re-estimations on correlation coefficients since meta-analyses could not be implemented in terms of Cohen's d and η^2 due to a lack of preprocessed data. While the heterogeneity estimates for the three studies mentioned above (in Cohen's d units) are accurately reproducible, estimates for all remaining meta-analyses differ quantitatively to some extent (less than 5 percentage points in terms of I^2 estimates) but are qualitatively akin.

Klein et al. (2014)[12]. *ManyLabs1* comprises multi-site replications of 16 effects (from 12 original publications)[36–47] involving 34–35 data collections across 36 independent labs each. We include the 16 primary meta-analytic results in our review. For each of the studied effects, the paper reports Cochran's Q-test and I^2, but no confidence intervals around I^2. The original article points readers to the project's OSF repository (osf.io/wx7ck) for the original data and code; however, this repository does not contain the preprocessed data used for the meta-analyses. Yet, the Wiki of the 'Analysis Scripts' component includes a link to a GitHub repository, which includes the preprocessed data and the analysis scripts for the meta-analyses reported in the paper. As described in an erratum[48], the original article contained some errors. One of these errors pertains to the heterogeneity results

for the ‘allowed/forbidden' effect[37], which were flawed due to an error in the initial analysis code. The analysis scripts have been corrected, and our reanalysis for the effect matches the corrected statistics reported in the erratum. For another effect[47], there are discrepancies (not mentioned in the erratum): while the Q-statistic obtained from the original data and code matches the reported number in the paper, the I^2 statistic differs (20.1% in the paper vs. 28.1% obtained in a reanalysis using the original code). All remaining heterogeneity estimates were reproducible. However, there are very minor discrepancies in results here and there, likely due to typos and/or rounding errors. All our re-estimates coincide with what is reported by the original authors in the file heterogeneity.pdf available in the project's GitHub repository (containing the log outputs generated when running the analysis script provided in the same repository).

Klein et al. (2018)[13]. *ManyLabs2* comprises multi-site direct and conceptual replications of 28 effects (from 26 publications)[39,49–73] involving data collections across 41–66 independent sites each. The paper reports Cochran's Q-test, τ, and I^2 for each studied effect. The paper also reports 95% confidence intervals around I^2 but no confidence interval around τ. As per the paper, the data and codes are available at osf.io/8cd4r; the Wiki of the OSF page indicates that the authors are aware of some bugs and points toward a GitHub repository to obtain reproducible data and code. We used the data and scripts available in this GitHub repo. In our review, we include 25 of the 28 meta-analyses; for three studies[71–73], the meta-analyses could not be reproduced as the preprocessed data is missing. These 25 studies are consistent with the meta-analytic results reported in the summary spreadsheet (meta_analysis_wide.xlsx) available in ML2’s GitHub repo. For all studies included in our review, we only consider the meta-analyses on all samples without moderators. All but one Q-tests and I^2 statistics (including the 95% CI) could be reproduced based on the original data: for one study[49], the Q-statistic in the re-estimation ($Q(48) = 10.02$) differs from what is reported in the paper ($Q(48) = 15.33$), while the I^2 (and the 95% CI around I^2) is consistent. Furthermore, the τ estimates reported in the paper are erroneous, likely due to a “mix of rounding errors and sometimes erroneously copying τ^2,” as acknowledged by the authors in the “Bug Tracker” logs linked in the OSF repository’s Wiki. Indeed, our re-estimates of τ are consistent with the original authors’ results as reported in the summary spreadsheet (meta_analysis_wide.xlsx).

Klein et al. (2022)[14]. *ManyLabs4* comprises multi-site replications of the theory by Greenberg et al.[74,75], involving data collections across 17 labs. While ten of the labs collected data using independently created “in-house” protocols, seven labs conducted the experiment using the “author-advised protocol.” Since the variability in estimates for the “in-house” sample does not only pertain to population heterogeneity (but also involves potential design heterogeneity), we re-estimate heterogeneity using a random-effects meta-analysis for the “author-approved protocols” only (based on the seven labs that implemented this protocol; $n = 699$ participants in total). The primary results by Klein et al. comprise three meta-analyses based on three different sets of exclusion criteria; for our review, we only consider the results of “exclusion set 1.” As per the paper, the data and codes are available at osf.io/8ccnw; the Wiki of the OSF page recommends using the

data and codes provided via the project's GitHub repository instead. The paper reports Cochran's Q-test, but neither τ nor I^2, for a meta-analysis pooling both the "in-house" and the "author-advised protocols," as well as for a meta-analysis accounting for moderation effects of author-advised vs. in-house protocols; the paper does not report heterogeneity estimates based on a meta-analysis of the "author approved protocol" sample only. Yet, the meta-analytic results of all 17 estimates are computationally reproducible, just as the meta-analytic effect size estimate for the "author-approved protocol" sample.

2 Design Heterogeneity

To the best of our knowledge, there are only two studies[76,77] that allow for systematic variation in effect size estimates pertaining to the same research question while ruling out other sources of potential variation to isolate design heterogeneity. Both studies are included in our analysis of design heterogeneity.

Huber et al. (2023)[76]. The study reports the results of a meta-analysis of 45 research designs proposed by independent teams. The original data and analysis scripts are available at osf.io/fyme2. We only consider heterogeneity estimates for analytic approach B, which rules out analytical heterogeneity by applying the same analysis to all experimental designs. All estimates relevant to our analyses are reported in the original paper: Q-statistic, τ together with its 95% CI, and I^2 together with its 95% CI. As in the original article, τ^2 is estimated using the DerSimonian-Laird[78] estimator. All estimates reported in the original paper are computationally reproducible in *R*, despite the original analysis having been carried out in *Stata*.

Landy et al. (2020)[77]. The study reports the meta-analytic results on five independent hypotheses comprising 12–13 research designs proposed by independent research teams. Data for the main studies and replications were collected in two populations for each of the five hypotheses. The original data and analysis scripts are available at osf.io/9jzy4. We consider both the main studies (for which data was collected on *Amazon Mechanical Turk*) and the replications (for which data was collected via *PureProfile*). All estimates relevant to our review (Q-test, τ^2 together with its 95% CI, and I^2 together with its 95% CI) are reported in the paper. We re-estimated the meta-analyses for the five hypotheses using the original data and codes. The regenerated results differ quantitatively from the results reported in Table 2 of the original paper, yet they are qualitatively similar. For hypothesis 5, the meta-analytic results reported in the paper pertain to correlation coefficients, but the original study's replication kit also includes a meta-analysis based on effect sizes transformed into Cohen's d units; for our analysis, we rely on the latter such that all meta-analytical estimates for the study are in Cohen's d units.

3 Analytical Heterogeneity

To gauge the extent of analytical heterogeneity, we include three multi-analyst studies[79–81]. Note that none of the multi-analyst studies included in our review report the results of a random-effects meta-analysis since estimating meta-analytic models on multi-analyst studies is unconventional (we refer to the main text for a discussion).

Hoogeveen et al. (2022)[79]. The study reports the results of 120 independent analysts using the same dataset to test two empirical claims. The original data and code are available at osf.io/bw863. As in the primary analysis of the paper, we only consider the n = 99 and n = 101 results for research questions 1 and 2, respectively, that are reported in terms of standardized β coefficients.

Huntington-Klein et al. (2021)[80]. The study reports the results of seven independent analysts on two causal empirical relationships. The original data and analysis scripts are available at osf.io/5637t. The original paper introduces the ratio between the standard deviation of effect size estimates across analysts and the mean standard error as a measure of analytical heterogeneity. We report the estimates of this alternative measure for all meta-analyses included in our review in Supplementary Tables S1 and S2.

Silberzahn et al. (2018)[81]. The study reports the results of 29 independent research teams using the same dataset to test the same hypothesis. The original data and code are available at osf.io/fa743. The data reports the effect size estimates per team (in terms of odds ratios) alongside the corresponding 95% CIs, but no standard errors. We transform the point estimate into log-odds ratios and back out standard errors by first log-transforming the upper/lower bounds of the 95% CI and then calculating the standard errors by dividing the (absolute) difference between the effect size estimate and the lower/upper bound of the 95% CI by the critical value of the normal distribution.

Supplementary references

1. Alogna, V. K. *et al.* Registered Replication Report: Schooler and Engstler-Schooler (1990). *Perspect. Psychol. Sci.* 9, 556–578 (2014).
2. Bouwmeester, S. *et al.* Registered Replication Report: Rand, Greene, and Nowak (2012). *Perspect. Psychol. Sci.* 12, 527–542 (2017).
3. Cheung, I. *et al.* Registered Replication Report: Study 1 From Finkel, Rusbult, Kumashiro, & Hannon (2002). *Perspect. Psychol. Sci.* 11, 750–764 (2016).
4. Eerland, A. *et al.* Registered Replication Report: Hart & Albarracín (2011). *Perspect. Psychol. Sci.* 11, 158–171 (2016).
5. Hagger, M. S. *et al.* A multilab preregistered replication of the ego-depletion effect. *Perspect. Psychol. Sci.* 11, 546–573 (2016).
6. McCarthy, R. J. *et al.* Registered Replication Report on Srull and Wyer (1979). *Adv. Methods Pract. Psychol. Sci.* 1, 321–336 (2018).
7. O'Donnell, M. *et al.* Registered Replication Report: Dijksterhuis and van Knippenberg (1998). *Perspect. Psychol. Sci.* 13, 268–294 (2018).
8. Verschuere, B. *et al.* Registered Replication Report on Mazar, Amir, and Ariely (2008). *Adv. Methods Pract. Psychol. Sci.* 1, 299–317 (2018).
9. Wagenmakers, E.-J. *et al.* Registered Replication Report: Strack, Martin, & Stepper (1988). *Perspect. Psychol. Sci.* 11, 917–928 (2016).
10. Colling, L. J. *et al.* Registered Replication Report on Fischer, Castel, Dodd, and Pratt (2003). *Adv. Methods Pract. Psychol. Sci.* 3, 143–162 (2020).
11. Ebersole, C. R. *et al.* Many Labs 3: Evaluating participant pool quality across the academic semester via replication. *J. Exp. Soc. Psychol.* 67, 68–82 (2016).
12. Klein, R. A. *et al.* Investigating variation in replicability: A "many labs" replication project. *Soc. Psychol.* 45, 142–152 (2014).
13. Klein, R. A. *et al.* Many Labs 2: Investigating variation in replicability across samples and settings. *Adv. Methods Pract. Psychol. Sci.* 1, 443–490 (2018).
14. Klein, R. A. *et al.* Many Labs 4: Failure to replicate mortality salience effect with and without original author involvement. *Collabra Psychol.* 8, 35271 (2022).
15. Ebersole, C. R. *et al.* Many Labs 5: Testing pre-data-collection peer review as an intervention to increase replicability. *Adv. Methods Pract. Psychol. Sci.* 3, 309–331 (2020).
16. Schooler, J. W. & Engstler-Schooler, T. Y. Verbal overshadowing of visual memories: Some things are better left unsaid. *Cognit. Psychol.* 22, 36–71 (1990).
17. Rand, D. G., Greene, J. D. & Nowak, M. A. Spontaneous giving and calculated greed. *Nature* 489, 427–430 (2012).
18. Finkel, E. J., Rusbult, C. E., Kumashiro, M. & Hannon, P. A. Dealing with betrayal in close relationships: Does commitment promote forgiveness? *J. Pers. Soc. Psychol.* 82, 956–974 (2002).

19. Hartung, J. & Knapp, G. On tests of the overall treatment effect in meta-analysis with normally distributed responses. *Stat. Med.* 20, 1771–1782 (2001).

20. Hart, W. & Albarracín, D. Learning about what others were doing: Verb aspect and attributions of mundane and criminal intent for past actions. *Psychol. Sci.* 22, 261–266 (2011).

21. Sripada, C., Kessler, D. & Jonides, J. Methylphenidate blocks effort-induced depletion of regulatory control in healthy volunteers. *Psychol. Sci.* 25, 1227–1234 (2014).

22. Srull, T. K. & Wyer, R. S. The role of category accessibility in the interpretation of information about persons: Some determinants and implications. *J. Pers. Soc. Psychol.* 37, 1660–1672 (1979).

23. Dijksterhuis, A. & van Knippenberg, A. The relation between perception and behavior, or how to win a game of Trivial Pursuit. *J. Pers. Soc. Psychol.* 74, 865–877 (1998).

24. Mazar, N., Amir, O. & Ariely, D. The dishonesty of honest people: A theory of self-concept maintenance. *J. Mark. Res.* 45, 633–644 (2008).

25. Strack, F., Martin, L. L. & Stepper, S. Inhibiting and facilitating conditions of the human smile: A nonobtrusive test of the facial feedback hypothesis. *J. Pers. Soc. Psychol.* 54, 768–777 (1988).

26. Tversky, A. & Kahneman, D. Availability: A heuristic for judging frequency and probability. *Cognit. Psychol.* 5, 207–232 (1973).

27. Stroop, J. R. Studies of interference in serial verbal reactions. *J. Exp. Psychol.* 18, 643–662 (1935).

28. Cacioppo, J. T., Petty, R. E. & Morris, K. J. Effects of need for cognition on message evaluation, recall, and persuasion. *J. Pers. Soc. Psychol.* 45, 805–818 (1983).

29. Boroditsky, L. Metaphoric structuring: Understanding time through spatial metaphors. *Cognition* 75, 1–28 (2000).

30. De Fruyt, F., Van De Wiele, L. & Van Heeringen, C. Cloninger's psychobiological model of temperament and character and the five-factor model of personality. *Personal. Individ. Differ.* 29, 441–452 (2000).

31. Monin, B. & Miller, D. T. Moral credentials and the expression of prejudice. *J. Pers. Soc. Psychol.* 81, 33–43 (2001).

32. Ross, M. & Wilson, A. E. It feels like yesterday: Self-esteem, valence of personal past experiences, and judgments of subjective distance. *J. Pers. Soc. Psychol.* 82, 792–803 (2002).

33. Galinsky, A. D., Magee, J. C., Inesi, M. E. & Gruenfeld, D. H. Power and perspectives not taken. *Psychol. Sci.* 17, 1068–1074 (2006).

34. Jostmann, N. B., Lakens, D. & Schubert, T. W. Weight as an embodiment of importance. *Psychol. Sci.* 20, 1169–1174 (2009).

35. Szymkow, A., Chandler, J., IJzerman, H., Parzuchowski, M. & Wojciszke, B. Warmer hearts, warmer rooms. *Soc. Psychol.* 44, 167–176 (2013).

36. Lorge, I. & Curtiss, C. C. Prestige, suggestion, and attitudes. *J. Soc. Psychol.* 7, 386–402 (1936).

37. Rugg, D. Experiments in wording questions II. *Public Opin. Q.* 5, 91–92 (1941).

38. Hyman, H. H. & Sheatsley, P. B. The current status of American public opinion. in *The teaching of contemporary affairs: 21st yearbook of the National Council of Social Studies* (ed. Payne, J. C.) 11–34

(National Council of Social Studies, 1950).

39. Tversky, A. & Kahneman, D. The framing of decisions and the psychology of choice. *Science* 211, 453–458 (1981).

40. Schwarz, N., Hippler, H.-J., Deutsch, B. & Strack, F. Response scales: Effects of category range on reported behavior and comparative judgments. *Public Opin. Q.* 49, 388–395 (1985).

41. Jacowitz, K. E. & Kahneman, D. Measures of anchoring in estimation tasks. *Pers. Soc. Psychol. Bull.* 21, 1161–1166 (1995).

42. Oppenheimer, D. M., Meyvis, T. & Davidenko, N. Instructional manipulation checks: Detecting satisficing to increase statistical power. *J. Exp. Soc. Psychol.* 45, 867–872 (2009).

43. Oppenheimer, D. M. & Monin, B. The retrospective gambler's fallacy: Unlikely events, constructing the past, and multiple universes. *Judgm. Decis. Mak.* 4, 326–334 (2009).

44. Husnu, S. & Crisp, R. J. Elaboration enhances the imagined contact effect. *J. Exp. Soc. Psychol.* 46, 943–950 (2010).

45. Carter, T. J., Ferguson, M. J. & Hassin, R. R. A single exposure to the American flag shifts support toward republicanism up to 8 months later. *Psychol. Sci.* 22, 1011–1018 (2011).

46. Caruso, E. M., Vohs, K. D., Baxter, B. & Waytz, A. Mere exposure to money increases endorsement of free-market systems and social inequality. *J. Exp. Psychol. Gen.* 142, 301–306 (2013).

47. Nosek, B. A., Banaji, M. R. & Greenwald, A. G. Math = male, me = female, therefore math ≠ me. *J. Pers. Soc. Psychol.* 83, 44–59 (2002).

48. Correction to Klein et al. (2014). *Soc. Psychol.* 50, 211–213 (2019).

49. Tversky, A. & Gati, I. Studies of similarity. *Cogn. Categ.* 79–98 (1978).

50. Shafir, E. Choosing versus rejecting: Why some options are both better and worse than others. *Mem. Cognit.* 21, 546–556 (1993).

51. Van Lange, P. A. M., De Bruin, E. M. N., Otten, W. & Joireman, J. A. Development of prosocial, individualistic, and competitive orientations: Theory and preliminary evidence. *J. Pers. Soc. Psychol.* 73, 733–746 (1997).

52. Hsee, C. K. Less is better: When low-value options are valued more highly than high-value options. *J. Behav. Decis. Mak.* 11, 107–121 (1998).

53. Rottenstreich, Y. & Hsee, C. K. Money, kisses, and electric shocks: On the affective psychology of risk. *Psychol. Sci.* 12, 185–190 (2001).

54. Miyamoto, Y. & Kitayama, S. Cultural variation in correspondence bias: the critical role of attitude diagnosticity of socially constrained behavior. *J. Pers. Soc. Psychol.* 83, 1239–1248 (2002).

55. Norenzayan, A., Smith, E. E., Kim, B. J. & Nisbett, R. E. Cultural preferences for formal versus intuitive reasoning. *Cogn. Sci.* 26, 653–684 (2002).

56. Knobe, J. The concept of intentional action: A case study in the uses of folk psychology. *Philos. Stud.* 130, 203–231 (2006).

57. Zhong, C.-B. & Liljenquist, K. Washing away your sins: Threatened morality and physical cleansing. *Science* 313, 1451–1452 (2006).

58. Alter, A. L., Oppenheimer, D. M., Epley, N. & Eyre, R. N. Overcoming intuition: Metacognitive difficulty activates analytic reasoning. *J. Exp. Psychol. Gen.* 136, 569–576 (2007).

59. Giessner, S. R. & Schubert, T. W. High in the hierarchy: How vertical location and judgments of leaders' power are interrelated. *Organ. Behav. Hum. Decis. Process.* 104, 30–44 (2007).

60. Hauser, M., Cushman, F., Young, L., Jin, R. K.-X. & Mikhail, J. A dissociation between moral judgments and justifications. *Mind Lang.* 22, 1–21 (2007).

61. Ross, L., Greene, D. & House, P. The false consensus effect: An egocentric bias in social perception and attribution processes. *J. Exp. Soc. Psychol.* 13, 279–301 (1977).

62. Critcher, C. R. & Gilovich, T. Incidental environmental anchors. *J. Behav. Decis. Mak.* 21, 241–251 (2008).

63. Risen, J. L. & Gilovich, T. Why people are reluctant to tempt fate. *J. Pers. Soc. Psychol.* 95, 293–307 (2008).

64. Gray, K. & Wegner, D. M. Moral typecasting: Divergent perceptions of moral agents and moral patients. *J. Pers. Soc. Psychol.* 96, 505–520 (2009).

65. Savani, K., Markus, H. R., Naidu, N. V. R., Kumar, S. & Berlia, N. What counts as a choice? U.S. Americans are more likely than Indians to construe actions as choices. *Psychol. Sci.* 21, 391–398 (2010).

66. Anderson, C., Kraus, M. W., Galinsky, A. D. & Keltner, D. The local-ladder effect: Social status and subjective well-being. *Psychol. Sci.* 23, 764–771 (2012).

67. Bauer, M. A., Wilkie, J. E. B., Kim, J. K. & Bodenhausen, G. V. Cuing consumerism: Situational materialism undermines personal and social well-being. *Psychol. Sci.* 23, 517–523 (2012).

68. Huang, Y., Tse, C.-S. & Cho, K. W. Living in the north is not necessarily favorable: Different metaphoric associations between cardinal direction and valence in Hong Kong and in the United States. *Eur. J. Soc. Psychol.* 44, 360–369 (2014).

69. Kay, A. C., Laurin, K., Fitzsimons, G. M. & Landau, M. J. A functional basis for structure-seeking: Exposure to structure promotes willingness to engage in motivated action. *J. Exp. Psychol. Gen.* 143, 486–491 (2014).

70. Zaval, L., Keenan, E. A., Johnson, E. J. & Weber, E. U. How warm days increase belief in global warming. *Nat. Clim. Change* 4, 143–147 (2014).

71. Schwarz, N., Strack, F. & Mai, H.-P. Assimilation and contrast effects in part-whole question sequences: A conversational logic analysis. *Public Opin. Q.* 55, 3–23 (1991).

72. Graham, J., Haidt, J. & Nosek, B. A. Liberals and conservatives rely on different sets of moral foundations. *J. Pers. Soc. Psychol.* 96, 1029–1046 (2009).

73. Inbar, Y., Pizarro, D. A., Knobe, J. & Bloom, P. Disgust sensitivity predicts intuitive disapproval of gays. *Emotion* 9, 435–439 (2009).

74. Greenberg, J., Pyszczynski, T. & Solomon, S. The causes and consequences of a need for self-esteem: A terror management theory. in *Public Self and Private Self* (ed. Baumeister, R. F.) 189–212 (Springer, 1986).

75. Greenberg, J., Pyszczynski, T., Solomon, S., Simon, L. & Breus, M. Role of consciousness and accessibility of death-related thoughts in mortality salience effects. *J. Pers. Soc. Psychol.* 67, 627–637 (1994).

76. Huber, C. *et al.* Competition and moral behavior: A meta-analysis of forty-five crowd-sourced experimental designs. *Proc. Natl. Acad. Sci.* 120, e2215572120 (2023).

77. Landy, J. F. *et al.* Crowdsourcing hypothesis tests: Making transparent how design choices shape research results. *Psychol. Bull.* 146, 451–479 (2020).

78. DerSimonian, R. & Laird, N. Meta-analysis in clinical trials. *Control. Clin. Trials* 7, 177–188 (1986).

79. Hoogeveen, S. *et al.* A many-analysts approach to the relation between religiosity and well-being. *Relig. Brain Behav.* 0, 1–47 (2022).

80. Huntington-Klein, N. *et al.* The influence of hidden researcher decisions in applied microeconomics. *Econ. Inq.* 59, 944–960 (2021).

81. Silberzahn, R. *et al.* Many analysts, one data set: Making transparent how variations in analytic choices affect results. *Adv. Methods Pract. Psychol. Sci.* 1, 337–356 (2018).

Supporting Tables

Table S1. Heterogeneity estimates. For each random-effects meta-analysis included in our review, the table reports the result of Cochran's Q-test (with Q being χ^2-distributed with k−1 degrees of freedom), whether or the effect sizes in the meta-analysis are measured in Cohen's d units, and various measures of between-study variation. As to the latter, we report τ (i.e., the estimated standard deviation of the underlying distribution of genuine effects) and the corresponding 95% confidence interval (CI) obtained using the Q-profile method, the I^2 statistic (indicating how much of the total variation is attributable to heterogeneity rather than sampling errors) and the corresponding 95% CI, and H (i.e., by how much the total variability in effect sizes, measured in standard deviation units, exceeds the sampling variation) and the corresponding 95% CI. Moreover, we report the meta-analyses' estimate of the within-study variation (in standard deviation units), σ, and an alternative measure of relative heterogeneity, HR (heterogeneity ratio), defined as the ratio of τ and σ.

**Type/Source**	**Study/Paper**	**Primary Study/Hypothesis**	***Q*-Test**	***d***	**Heterogeneity [illegible]**	**[illegible]**	***HR***	**Heterogeneity I^2**	**Heterogeneity *H***
Population Heterogeneity	Alogna et al. (2014)[1]	Schooler & Engstler-Schooler (1990)[16]: Study 4	$Q(30) = 29.3$ $p = 0.501$	☒	$\tau = 0.000$ $CI_{95\%}$ [0.000, 0.077]	0.089	0.000	$I^2 = 0.0$ $CI_{95\%}$ [0.0, 42.9]	$H = 1.000$ $CI_{95\%}$ [1.000, 1.324]
Population Heterogeneity	Alogna et al. (2014)[1]	Schooler & Engstler-Schooler (1990)[16]: Study 1	$Q(21) = 15.3$ $p = 0.807$	☒	$\tau = 0.000$ $CI_{95\%}$ [0.000, 0.069]	0.100	0.000	$I^2 = 0.0$ $CI_{95\%}$ [0.0, 32.3]	$H = 1.000$ $CI_{95\%}$ [1.000, 1.216]
Population Heterogeneity	Bouwmeester et al. (2017)[2]	Rand, Greene, & Nowak (2012)[17]	$Q(20) = 16.8$ $p = 0.664$	☒	$\tau = 0.897$ $CI_{95\%}$ [0.000, 4.341]	5.367	0.167	$I^2 = 2.7$ $CI_{95\%}$ [0.0, 39.5]	$H = 1.014$ $CI_{95\%}$ [1.000, 1.286]
Population Heterogeneity	Cheung et al. (2016)[3]	Finkel et al. (2012)[18]: Subjective commitment	$Q(15) = 5.3$ $p = 0.989$	☒	$\tau = 0.000$ $CI_{95\%}$ [0.000, 0.000]	0.264	0.000	$I^2 = 0.0$ $CI_{95\%}$ [0.0, 0.0]	$H = 1.000$ $CI_{95\%}$ [1.000, 1.000]
Population Heterogeneity	Cheung et al. (2016)[3]	Finkel et al. (2012)[18]: Exit forgiveness	$Q(15) = 14.4$ $p = 0.498$	☒	$\tau = 0.032$ $CI_{95\%}$ [0.000, 0.237]	0.213	0.151	$I^2 = 2.2$ $CI_{95\%}$ [0.0, 55.3]	$H = 1.011$ $CI_{95\%}$ [1.000, 1.496]
Population Heterogeneity	Cheung et al. (2016)[3]	Finkel et al. (2012)[18]: Neglect forgiveness	$Q(15) = 18.5$ $p = 0.239$	☒	$\tau = 0.107$ $CI_{95\%}$ [0.000, 0.298]	0.214	0.501	$I^2 = 20.1$ $CI_{95\%}$ [0.0, 66.0]	$H = 1.119$ $CI_{95\%}$ [1.000, 1.714]
Population Heterogeneity	Cheung et al. (2016)[3]	Finkel et al. (2012)[18]: Voice forgiveness	$Q(15) = 11.4$ $p = 0.726$	☒	$\tau = 0.000$ $CI_{95\%}$ [0.000, 0.206]	0.230	0.000	$I^2 = 0.0$ $CI_{95\%}$ [0.0, 44.5]	$H = 1.000$ $CI_{95\%}$ [1.000, 1.342]
Population Heterogeneity	Cheung et al. (2016)[3]	Finkel et al. (2012)[18]: Loyalty forgiveness	$Q(15) = 9.0$ $p = 0.878$	☒	$\tau = 0.000$ $CI_{95\%}$ [0.000, 0.144]	0.209	0.000	$I^2 = 0.0$ $CI_{95\%}$ [0.0, 32.3]	$H = 1.000$ $CI_{95\%}$ [1.000, 1.215]
Population Heterogeneity	Eerland et al. (2016)[4]	Hart & Albarracín (2011)[20]: Intentionality	$Q(10) = 18.6$ $p = 0.045$	☒	$\tau = 0.291$ $CI_{95\%}$ [0.000, 0.782]	0.322	0.903	$I^2 = 44.9$ $CI_{95\%}$ [0.0, 85.5]	$H = 1.348$ $CI_{95\%}$ [1.000, 2.628]
Population Heterogeneity	Eerland et al. (2016)[4]	Hart & Albarracín (2011)[20]: Imagery	$Q(10) = 7.7$ $p = 0.662$	☒	$\tau = 0.000$ $CI_{95\%}$ [0.000, 0.284]	0.249	0.000	$I^2 = 0.0$ $CI_{95\%}$ [0.0, 56.4]	$H = 1.000$ $CI_{95\%}$ [1.000, 1.515]

cont'd on next page

Type/Source	Study/Paper	Primary Study/Hypothesis	*Q*-Test	*d*	Heterogeneity [illegible]	[illegible]	*HR*	Heterogeneity I^2	Heterogeneity *H*
Population Heterogeneity	Eerland et al. (2016)[4]	Hart & Albarracín (2011)[20]: Intention Attribution	$Q(10) = 10.6$<br>$p = 0.393$	☒	$\tau = 0.002$<br>$CI_{95\%}$ [0.000, 0.209]	0.128	0.014	$I^2 = 0.0$<br>$CI_{95\%}$ [0.0, 72.6]	$H = 1.000$<br>$CI_{95\%}$ [1.000, 1.911]
Population Heterogeneity	Hagger et al. (2016)[5]	Sripada et al. (2014)[21]: Reaction Time Variability	$Q(22) = 33.8$<br>$p = 0.052$	☑	$\tau = 0.143$<br>$CI_{95\%}$ [0.000, 0.326]	0.209	0.682	$I^2 = 31.7$<br>$CI_{95\%}$ [0.0, 70.8]	$H = 1.210$<br>$CI_{95\%}$ [1.000, 1.852]
Population Heterogeneity	Hagger et al. (2016)[5]	Sripada et al. (2014)[21]: Reaction Times	$Q(22) = 32.8$<br>$p = 0.065$	☑	$\tau = 0.131$<br>$CI_{95\%}$ [0.000, 0.323]	0.209	0.626	$I^2 = 28.1$<br>$CI_{95\%}$ [0.0, 70.4]	$H = 1.180$<br>$CI_{95\%}$ [1.000, 1.837]
Population Heterogeneity	McCarthy et al. (2018)[6]	Srull & Wyer (1979)[22]: Ronald's hostility	$Q(21) = 25.3$<br>$p = 0.234$	☒	$\tau = 0.079$<br>$CI_{95\%}$ [0.000, 0.207]	0.170	0.464	$I^2 = 17.7$<br>$CI_{95\%}$ [0.0, 59.6]	$H = 1.102$<br>$CI_{95\%}$ [1.000, 1.574]
Population Heterogeneity	McCarthy et al. (2018)[6]	Srull & Wyer (1979)[22]: Hostile behaviors	$Q(21) = 24.4$<br>$p = 0.275$	☒	$\tau = 0.096$<br>$CI_{95\%}$ [0.000, 0.233]	0.206	0.469	$I^2 = 18.0$<br>$CI_{95\%}$ [0.0, 56.3]	$H = 1.104$<br>$CI_{95\%}$ [1.000, 1.512]
Population Heterogeneity	O'Donnell et al. (2018)[7]	Dijksterhuis & van Knippenberg (1998)[23]	$Q(22) = 28.1$<br>$p = 0.173$	☒	$\tau = 0.860$<br>$CI_{95\%}$ [0.000, 2.540]	1.872	0.459	$I^2 = 17.4$<br>$CI_{95\%}$ [0.0, 64.8]	$H = 1.101$<br>$CI_{95\%}$ [1.000, 1.685]
Population Heterogeneity	Verschuere et al. (2018)[8]	Mazar, Amir, & Ariely (2008)[24]	$Q(18) = 13.2$<br>$p = 0.782$	☒	$\tau = 0.000$<br>$CI_{95\%}$ [0.000, 0.392]	0.441	0.000	$I^2 = 0.0$<br>$CI_{95\%}$ [0.0, 44.1]	$H = 1.000$<br>$CI_{95\%}$ [1.000, 1.338]
Population Heterogeneity	Wagenmakers et al. (2016)[9]	Strack, Martin, & Stepper (1988)[25]	$Q(16) = 9.0$<br>$p = 0.915$	☒	$\tau = 0.000$<br>$CI_{95\%}$ [0.000, 0.162]	0.283	0.000	$I^2 = 0.0$<br>$CI_{95\%}$ [0.0, 24.7]	$H = 1.000$<br>$CI_{95\%}$ [1.000, 1.152]
Population Heterogeneity	Ebersole et al. (2016)[11]	Tversky & Kahneman (1973)[26]	$Q(20) = 19.8$<br>$p = 0.473$	☒	$\tau = 0.006$<br>$CI_{95\%}$ [0.000, 0.095]	0.084	0.072	$I^2 = 0.5$<br>$CI_{95\%}$ [0.0, 56.1]	$H = 1.003$<br>$CI_{95\%}$ [1.000, 1.509]
Population Heterogeneity	Ebersole et al. (2016)[11]	Stroop (1935)[27]	$Q(20) = 11.6$<br>$p = 0.929$	☒	$\tau = 0.000$<br>$CI_{95\%}$ [0.000, 0.027]	0.068	0.000	$I^2 = 0.0$<br>$CI_{95\%}$ [0.0, 13.6]	$H = 1.000$<br>$CI_{95\%}$ [1.000, 1.076]
Population Heterogeneity	Ebersole et al. (2016)[11]	Cacioppo et al. (1983)[28]	$Q(19) = 11.4$<br>$p = 0.911$	☒	$\tau = 0.000$<br>$CI_{95\%}$ [0.000, 0.042]	0.088	0.000	$I^2 = 0.0$<br>$CI_{95\%}$ [0.0, 18.6]	$H = 1.000$<br>$CI_{95\%}$ [1.000, 1.109]
Population Heterogeneity	Ebersole et al. (2016)[11]	Boroditsky (2000)[29]	$Q(19) = 20.8$<br>$p = 0.349$	☒	$\tau = 0.047$<br>$CI_{95\%}$ [0.000, 0.141]	0.123	0.387	$I^2 = 13.0$<br>$CI_{95\%}$ [0.0, 57.0]	$H = 1.072$<br>$CI_{95\%}$ [1.000, 1.525]
Population Heterogeneity	Ebersole et al. (2016)[11]	DeFruyt et al. (2000)[30]	$Q(20) = 22.0$<br>$p = 0.341$	☒	$\tau = 0.000$<br>$CI_{95\%}$ [0.000, 0.104]	0.083	0.002	$I^2 = 0.0$<br>$CI_{95\%}$ [0.0, 61.4]	$H = 1.000$<br>$CI_{95\%}$ [1.000, 1.610]
Population Heterogeneity	Ebersole et al. (2016)[11]	Monin & Miller (2001)[31]	$Q(20) = 29.3$<br>$p = 0.082$	☒	$\tau = 0.046$<br>$CI_{95\%}$ [0.000, 0.137]	0.082	0.562	$I^2 = 24.0$<br>$CI_{95\%}$ [0.0, 73.8]	$H = 1.147$<br>$CI_{95\%}$ [1.000, 1.954]
Population Heterogeneity	Ebersole et al. (2016)[11]	Ross & Wilson (2002)[32]	$Q(20) = 32.2$<br>$p = 0.041$	☒	$\tau = 0.059$<br>$CI_{95\%}$ [0.000, 0.151]	0.083	0.710	$I^2 = 33.5$<br>$CI_{95\%}$ [0.0, 76.8]	$H = 1.226$<br>$CI_{95\%}$ [1.000, 2.075]
Population Heterogeneity	Ebersole et al. (2016)[11]	Galinsky et al. (2006)[33]	$Q(20) = 19.8$<br>$p = 0.471$	☑	$\tau = 0.002$<br>$CI_{95\%}$ [0.000, 0.198]	0.171	0.009	$I^2 = 0.0$<br>$CI_{95\%}$ [0.0, 57.2]	$H = 1.000$<br>$CI_{95\%}$ [1.000, 1.528]

cont'd on next page

Type/Source	Study/Paper	Primary Study/Hypothesis	*Q*-Test	*d*	Heterogeneity [illegible]	[illegible]	*HR*	Heterogeneity I^2	Heterogeneity *H*
Population Heterogeneity	Ebersole et al. (2016)[11]	Jostman et al. (2009)[34]	$Q(19) = 12.3$<br>$p = 0.875$	☑	$\tau = 0.000$<br>$CI_{95\%}$ [0.000, 0.122]	0.187	0.000	$I^2 = 0.0$<br>$CI_{95\%}$ [0.0, 30.0]	$H = 1.000$<br>$CI_{95\%}$ [1.000, 1.195]
Population Heterogeneity	Ebersole et al. (2016)[11]	Szymkow et al. (2013)[35]	$Q(20) = 16.9$<br>$p = 0.657$	☑	$\tau = 0.000$<br>$CI_{95\%}$ [0.000, 0.158]	0.168	0.000	$I^2 = 0.0$<br>$CI_{95\%}$ [0.0, 47.1]	$H = 1.000$<br>$CI_{95\%}$ [1.000, 1.375]
Population Heterogeneity	Klein et al. (2014)[12]	Lorge & Curtiss (1936)[36]	$Q(35) = 67.7$<br>$p = 0.001$	☑	$\tau = 0.164$<br>$CI_{95\%}$ [0.090, 0.282]	0.157	1.042	$I^2 = 52.0$<br>$CI_{95\%}$ [24.6, 76.3]	$H = 1.444$<br>$CI_{95\%}$ [1.152, 2.052]
Population Heterogeneity	Klein et al. (2014)[12]	Rugg (1941)[37]	$Q(35) = 29.0$<br>$p = 0.754$	☑	$\tau = 0.000$<br>$CI_{95\%}$ [0.000, 0.198]	0.282	0.000	$I^2 = 0.0$<br>$CI_{95\%}$ [0.0, 33.0]	$H = 1.000$<br>$CI_{95\%}$ [1.000, 1.222]
Population Heterogeneity	Klein et al. (2014)[12]	Hyman & Sheatsley (1950)[38]	$Q(35) = 38.9$<br>$p = 0.299$	☑	$\tau = 0.091$<br>$CI_{95\%}$ [0.000, 0.190]	0.200	0.456	$I^2 = 17.2$<br>$CI_{95\%}$ [0.0, 47.5]	$H = 1.099$<br>$CI_{95\%}$ [1.000, 1.380]
Population Heterogeneity	Klein et al. (2014)[12]	Tversky & Kahneman (1981)[39]	$Q(35) = 37.0$<br>$p = 0.376$	☑	$\tau = 0.002$<br>$CI_{95\%}$ [0.000, 0.205]	0.183	0.010	$I^2 = 0.0$<br>$CI_{95\%}$ [0.0, 55.6]	$H = 1.000$<br>$CI_{95\%}$ [1.000, 1.500]
Population Heterogeneity	Klein et al. (2014)[12]	Schwarz et al. (1985)[40]	$Q(35) = 36.0$<br>$p = 0.421$	☑	$\tau = 0.155$<br>$CI_{95\%}$ [0.000, 0.318]	0.319	0.487	$I^2 = 19.2$<br>$CI_{95\%}$ [0.0, 50.0]	$H = 1.112$<br>$CI_{95\%}$ [1.000, 1.414]
Population Heterogeneity	Klein et al. (2014)[12]	Jacowitz & Kahneman (1995)[41]: Distance to NYC	$Q(35) = 59.7$<br>$p = 0.006$	☑	$\tau = 0.152$<br>$CI_{95\%}$ [0.064, 0.311]	0.185	0.820	$I^2 = 40.2$<br>$CI_{95\%}$ [10.6, 73.9]	$H = 1.293$<br>$CI_{95\%}$ [1.058, 1.959]
Population Heterogeneity	Klein et al. (2014)[12]	Jacowitz & Kahneman (1995)[41]: Population of Chicago	$Q(35) = 152.3$<br>$p < 0.001$	☑	$\tau = 0.358$<br>$CI_{95\%}$ [0.257, 0.533]	0.205	1.749	$I^2 = 75.4$<br>$CI_{95\%}$ [61.1, 87.1]	$H = 2.014$<br>$CI_{95\%}$ [1.604, 2.789]
Population Heterogeneity	Klein et al. (2014)[12]	Jacowitz & Kahneman (1995)[41]: Height of Mt. Everest	$Q(35) = 312.8$<br>$p < 0.001$	☑	$\tau = 0.693$<br>$CI_{95\%}$ [0.544, 0.956]	0.214	3.237	$I^2 = 91.3$<br>$CI_{95\%}$ [86.6, 95.2]	$H = 3.388$<br>$CI_{95\%}$ [2.733, 4.580]
Population Heterogeneity	Klein et al. (2014)[12]	Jacowitz & Kahneman (1995)[41]: Babies Born	$Q(35) = 88.1$<br>$p < 0.001$	☑	$\tau = 0.298$<br>$CI_{95\%}$ [0.202, 0.492]	0.220	1.353	$I^2 = 64.7$<br>$CI_{95\%}$ [45.7, 83.3]	$H = 1.682$<br>$CI_{95\%}$ [1.357, 2.449]
Population Heterogeneity	Klein et al. (2014)[12]	Oppenheimer et al. (2009)[42]	$Q(35) = 35.5$<br>$p = 0.442$	☑	$\tau = 0.050$<br>$CI_{95\%}$ [0.000, 0.145]	0.157	0.318	$I^2 = 9.2$<br>$CI_{95\%}$ [0.0, 45.9]	$H = 1.049$<br>$CI_{95\%}$ [1.000, 1.360]
Population Heterogeneity	Klein et al. (2014)[12]	Oppenheimer & Monin (2009)[43]	$Q(35) = 50.8$<br>$p = 0.041$	☑	$\tau = 0.090$<br>$CI_{95\%}$ [0.000, 0.248]	0.166	0.544	$I^2 = 22.8$<br>$CI_{95\%}$ [0.0, 69.2]	$H = 1.138$<br>$CI_{95\%}$ [1.000, 1.801]
Population Heterogeneity	Klein et al. (2014)[12]	Husnu & Crisp (2010)[44]	$Q(35) = 45.9$<br>$p = 0.103$	☑	$\tau = 0.080$<br>$CI_{95\%}$ [0.000, 0.202]	0.156	0.509	$I^2 = 20.6$<br>$CI_{95\%}$ [0.0, 62.5]	$H = 1.122$<br>$CI_{95\%}$ [1.000, 1.633]
Population Heterogeneity	Klein et al. (2014)[12]	Carter et al. (2011)[45]	$Q(35) = 30.3$<br>$p = 0.693$	☑	$\tau = 0.000$<br>$CI_{95\%}$ [0.000, 0.118]	0.157	0.000	$I^2 = 0.0$<br>$CI_{95\%}$ [0.0, 36.2]	$H = 1.000$<br>$CI_{95\%}$ [1.000, 1.252]
Population Heterogeneity	Klein et al. (2014)[12]	Caruso et al. (2013)[46]	$Q(35) = 28.4$<br>$p = 0.777$	☑	$\tau = 0.000$<br>$CI_{95\%}$ [0.000, 0.110]	0.156	0.000	$I^2 = 0.0$<br>$CI_{95\%}$ [0.0, 33.2]	$H = 1.000$<br>$CI_{95\%}$ [1.000, 1.223]

cont'd on next page

Type/Source	Study/Paper	Primary Study/Hypothesis	*Q*-Test	*d*	Heterogeneity [illegible]	[illegible]	*HR*	Heterogeneity I^2	Heterogeneity *H*
Population Heterogeneity	Klein et al. (2014)[12]	Nosek et al. (2002)[47]: Sex Differences	$Q(35) = 47.6$<br>$p = 0.061$	☑	$\tau = 0.112$<br>CI$_{95\%}$ [0.000, 0.258]	0.180	0.625	$I^2 = 28.1$<br>CI$_{95\%}$ [0.0, 67.3]	$H = 1.179$<br>CI$_{95\%}$ [1.000, 1.750]
Population Heterogeneity	Klein et al. (2014)[12]	Nosek et al. (2002)[47]: Math Attitudes	$Q(34) = 54.8$<br>$p = 0.013$	☑	$\tau = 0.056$<br>CI$_{95\%}$ [0.014, 0.094]	0.069	0.817	$I^2 = 40.0$<br>CI$_{95\%}$ [3.9, 65.0]	$H = 1.291$<br>CI$_{95\%}$ [1.020, 1.690]
Population Heterogeneity	Klein et al. (2018)[13]	Tversky & Kahneman (1981)[39]	$Q(54) = 55.2$<br>$p = 0.429$	☑	$\tau = 0.025$<br>CI$_{95\%}$ [0.000, 0.075]	0.099	0.251	$I^2 = 5.9$<br>CI$_{95\%}$ [0.0, 36.5]	$H = 1.031$<br>CI$_{95\%}$ [1.000, 1.255]
Population Heterogeneity	Klein et al. (2018)[13]	Tversky & Gati (1978)[49]	$Q(48) = 10.0$<br>$p = 1.000$	☑	$\tau = 0.000$<br>CI$_{95\%}$ [0.000, 0.000]	0.123	0.000	$I^2 = 0.0$<br>CI$_{95\%}$ [0.0, 0.0]	$H = 1.000$<br>CI$_{95\%}$ [1.000, 1.000]
Population Heterogeneity	Klein et al. (2018)[13]	Shafir (1993)[50]	$Q(40) = 51.7$<br>$p = 0.102$	☑	$\tau = 0.047$<br>CI$_{95\%}$ [0.000, 0.083]	0.079	0.600	$I^2 = 26.5$<br>CI$_{95\%}$ [0.0, 52.4]	$H = 1.166$<br>CI$_{95\%}$ [1.000, 1.450]
Population Heterogeneity	Klein et al. (2018)[13]	van Lange et al. (1997)[51]	$Q(53) = 103.6$<br>$p < 0.001$	☑	$\tau = 0.069$<br>CI$_{95\%}$ [0.043, 0.100]	0.069	1.004	$I^2 = 50.2$<br>CI$_{95\%}$ [28.2, 67.9]	$H = 1.417$<br>CI$_{95\%}$ [1.180, 1.765]
Population Heterogeneity	Klein et al. (2018)[13]	Hsee (1998)[52]	$Q(56) = 158.4$<br>$p < 0.001$	☑	$\tau = 0.099$<br>CI$_{95\%}$ [0.071, 0.133]	0.073	1.355	$I^2 = 64.7$<br>CI$_{95\%}$ [48.8, 77.0]	$H = 1.684$<br>CI$_{95\%}$ [1.398, 2.083]
Population Heterogeneity	Klein et al. (2018)[13]	Rottenstreich & Hsee (2001)[53]	$Q(59) = 50.8$<br>$p = 0.769$	☑	$\tau = 0.000$<br>CI$_{95\%}$ [0.000, 0.056]	0.108	0.000	$I^2 = 0.0$<br>CI$_{95\%}$ [0.0, 21.1]	$H = 1.000$<br>CI$_{95\%}$ [1.000, 1.126]
Population Heterogeneity	Klein et al. (2018)[13]	Miyamoto & Kitayama (2002)[54]	$Q(57) = 235.7$<br>$p < 0.001$	☑	$\tau = 0.064$<br>CI$_{95\%}$ [0.044, 0.078]	0.047	1.354	$I^2 = 64.7$<br>CI$_{95\%}$ [46.2, 73.1]	$H = 1.683$<br>CI$_{95\%}$ [1.363, 1.927]
Population Heterogeneity	Klein et al. (2018)[13]	Norenzayan et al. (2002)[55]	$Q(56) = 156.8$<br>$p < 0.001$	☑	$\tau = 0.103$<br>CI$_{95\%}$ [0.080, 0.150]	0.073	1.408	$I^2 = 66.5$<br>CI$_{95\%}$ [54.4, 80.9]	$H = 1.727$<br>CI$_{95\%}$ [1.480, 2.286]
Population Heterogeneity	Klein et al. (2018)[13]	Knobe (2003)[56]	$Q(58) = 631.7$<br>$p < 0.001$	☑	$\tau = 0.148$<br>CI$_{95\%}$ [0.129, 0.205]	0.039	3.784	$I^2 = 93.5$<br>CI$_{95\%}$ [91.7, 96.5]	$H = 3.914$<br>CI$_{95\%}$ [3.462, 5.354]
Population Heterogeneity	Klein et al. (2018)[13]	Zhong & Liljenquist (2006)[57]	$Q(51) = 65.6$<br>$p = 0.082$	☑	$\tau = 0.047$<br>CI$_{95\%}$ [0.000, 0.090]	0.087	0.536	$I^2 = 22.3$<br>CI$_{95\%}$ [0.0, 51.5]	$H = 1.134$<br>CI$_{95\%}$ [1.000, 1.437]
Population Heterogeneity	Klein et al. (2018)[13]	Alter et al. (2007)[58]	$Q(65) = 59.5$<br>$p = 0.671$	☑	$\tau = 0.001$<br>CI$_{95\%}$ [0.000, 0.061]	0.099	0.012	$I^2 = 0.0$<br>CI$_{95\%}$ [0.0, 27.4]	$H = 1.000$<br>CI$_{95\%}$ [1.000, 1.174]
Population Heterogeneity	Klein et al. (2018)[13]	Giessner & Schubert (2007)[59]	$Q(58) = 62.9$<br>$p = 0.308$	☑	$\tau = 0.016$<br>CI$_{95\%}$ [0.000, 0.074]	0.087	0.178	$I^2 = 3.1$<br>CI$_{95\%}$ [0.0, 42.2]	$H = 1.016$<br>CI$_{95\%}$ [1.000, 1.315]
Population Heterogeneity	Klein et al. (2018)[13]	Hauser et al. (2007)[60]: Scenarios 1 and 2	$Q(58) = 131.2$<br>$p < 0.001$	☑	$\tau = 0.080$<br>CI$_{95\%}$ [0.050, 0.102]	0.073	1.085	$I^2 = 54.1$<br>CI$_{95\%}$ [31.8, 66.2]	$H = 1.476$<br>CI$_{95\%}$ [1.211, 1.719]
Population Heterogeneity	Klein et al. (2018)[13]	Hauser et al. (2007)[60]: Scenarios 3 and 4	$Q(59) = 60.4$<br>$p = 0.425$	☑	$\tau = 0.036$<br>CI$_{95\%}$ [0.000, 0.069]	0.097	0.367	$I^2 = 11.9$<br>CI$_{95\%}$ [0.0, 33.2]	$H = 1.065$<br>CI$_{95\%}$ [1.000, 1.224]

cont'd on next page

Type/Source	Study/Paper	Primary Study/Hypothesis	*Q*-Test	*d*	Heterogeneity [illegible]	[illegible]	*HR*	Heterogeneity I^2	Heterogeneity *H*
Population Heterogeneity	Klein et al. (2018)[13]	Ross et al. (1977)[61]: Supermarket Scenario	$Q(58) = 65.5$<br>$p = 0.232$	☑	$\tau = 0.032$<br>$CI_{95\%}$ [0.000, 0.061]	0.073	0.434	$I^2 = 15.9$<br>$CI_{95\%}$ [0.0, 40.5]	$H = 1.090$<br>$CI_{95\%}$ [1.000, 1.297]
Population Heterogeneity	Klein et al. (2018)[13]	Ross et al. (1977)[61]: Traffic-Ticket Scenario	$Q(57) = 100.2$<br>$p < 0.001$	☑	$\tau = 0.063$<br>$CI_{95\%}$ [0.034, 0.093]	0.072	0.871	$I^2 = 43.2$<br>$CI_{95\%}$ [18.1, 62.6]	$H = 1.326$<br>$CI_{95\%}$ [1.105, 1.636]
Population Heterogeneity	Klein et al. (2018)[13]	Critcher & Gilovich (2008)[62]	$Q(58) = 64.9$<br>$p = 0.249$	☑	$\tau = 0.024$<br>$CI_{95\%}$ [0.000, 0.084]	0.097	0.243	$I^2 = 5.6$<br>$CI_{95\%}$ [0.0, 42.5]	$H = 1.029$<br>$CI_{95\%}$ [1.000, 1.319]
Population Heterogeneity	Klein et al. (2018)[13]	Risen & Gilovich (2008)[63]	$Q(58) = 87.8$<br>$p = 0.007$	☑	$\tau = 0.065$<br>$CI_{95\%}$ [0.021, 0.091]	0.085	0.758	$I^2 = 36.5$<br>$CI_{95\%}$ [5.9, 53.6]	$H = 1.255$<br>$CI_{95\%}$ [1.031, 1.468]
Population Heterogeneity	Klein et al. (2018)[13]	Gray & Wegner (2009)[64]	$Q(59) = 203.3$<br>$p < 0.001$	☑	$\tau = 0.110$<br>$CI_{95\%}$ [0.085, 0.147]	0.067	1.642	$I^2 = 72.9$<br>$CI_{95\%}$ [61.7, 82.8]	$H = 1.922$<br>$CI_{95\%}$ [1.616, 2.409]
Population Heterogeneity	Klein et al. (2018)[13]	Savani et al. (2010)[65]	$Q(56) = 155.5$<br>$p < 0.001$	☑	$\tau = 0.061$<br>$CI_{95\%}$ [0.043, 0.081]	0.046	1.331	$I^2 = 63.9$<br>$CI_{95\%}$ [46.8, 76.0]	$H = 1.664$<br>$CI_{95\%}$ [1.371, 2.040]
Population Heterogeneity	Klein et al. (2018)[13]	Anderson et al. (2012)[66]	$Q(58) = 55.1$<br>$p = 0.584$	☑	$\tau = 0.013$<br>$CI_{95\%}$ [0.000, 0.063]	0.097	0.137	$I^2 = 1.8$<br>$CI_{95\%}$ [0.0, 29.8]	$H = 1.009$<br>$CI_{95\%}$ [1.000, 1.194]
Population Heterogeneity	Klein et al. (2018)[13]	Bauer et al. (2012)[67]	$Q(53) = 63.8$<br>$p = 0.147$	☑	$\tau = 0.035$<br>$CI_{95\%}$ [0.000, 0.093]	0.094	0.369	$I^2 = 12.0$<br>$CI_{95\%}$ [0.0, 49.1]	$H = 1.066$<br>$CI_{95\%}$ [1.000, 1.402]
Population Heterogeneity	Klein et al. (2018)[13]	Huang et al. (2014)[68]	$Q(63) = 626.3$<br>$p < 0.001$	☑	$\tau = 0.247$<br>$CI_{95\%}$ [0.202, 0.301]	0.088	2.811	$I^2 = 88.8$<br>$CI_{95\%}$ [84.1, 92.2]	$H = 2.984$<br>$CI_{95\%}$ [2.511, 3.569]
Population Heterogeneity	Klein et al. (2018)[13]	Kay et al. (2014)[69]	$Q(51) = 34.0$<br>$p = 0.968$	☑	$\tau = 0.000$<br>$CI_{95\%}$ [0.000, 0.013]	0.095	0.000	$I^2 = 0.0$<br>$CI_{95\%}$ [0.0, 1.9]	$H = 1.000$<br>$CI_{95\%}$ [1.000, 1.010]
Population Heterogeneity	Klein et al. (2018)[13]	Zaval et al. (2014)[70]	$Q(46) = 73.0$<br>$p = 0.007$	☑	$\tau = 0.082$<br>$CI_{95\%}$ [0.032, 0.140]	0.108	0.762	$I^2 = 36.8$<br>$CI_{95\%}$ [8.2, 62.7]	$H = 1.257$<br>$CI_{95\%}$ [1.043, 1.638]
Population Heterogeneity	Klein et al. (2022)[14]	Greenberg et al. (1994)[75]	$Q(6) = 4.4$<br>$p = 0.622$	☒	$\tau = 0.000$<br>$CI_{95\%}$ [0.000, 0.334]	0.202	0.000	$I^2 = 0.0$<br>$CI_{95\%}$ [0.0, 73.2]	$H = 1.000$<br>$CI_{95\%}$ [1.000, 1.933]
Design Heterogeneity	Huber et al. (2023)[76]	Competition and Moral Behavior	$Q(44) = 161.5$<br>$p < 0.001$	☑	$\tau = 0.169$<br>$CI_{95\%}$ [0.140, 0.254]	0.103	1.634	$I^2 = 72.8$<br>$CI_{95\%}$ [64.8, 85.8]	$H = 1.916$<br>$CI_{95\%}$ [1.685, 2.657]
Design Heterogeneity	Landy et al. (2021)[77]	Awareness of Automatic Prejudice	$Q(12) = 892.5$<br>$p < 0.001$	☑	$\tau = 0.534$<br>$CI_{95\%}$ [0.380, 0.897]	0.051	10.388	$I^2 = 99.1$<br>$CI_{95\%}$ [98.2, 99.7]	$H = 10.436$<br>$CI_{95\%}$ [7.473, 17.496]
Design Heterogeneity	Landy et al. (2021)[77]	Awareness of Automatic Prejudice (Replication)	$Q(12) = 775.0$<br>$p < 0.001$	☑	$\tau = 0.477$<br>$CI_{95\%}$ [0.340, 0.797]	0.051	9.416	$I^2 = 98.9$<br>$CI_{95\%}$ [97.8, 99.6]	$H = 9.469$<br>$CI_{95\%}$ [6.783, 15.779]
Design Heterogeneity	Landy et al. (2021)[77]	Extreme Offers Reduce Trust	$Q(12) = 628.8$<br>$p < 0.001$	☑	$\tau = 0.783$<br>$CI_{95\%}$ [0.557, 1.306]	0.097	8.080	$I^2 = 98.5$<br>$CI_{95\%}$ [97.1, 99.5]	$H = 8.142$<br>$CI_{95\%}$ [5.834, 13.506]

cont'd on next page

Type/Source	Study/Paper	Primary Study/Hypothesis	Q-Test	d	Heterogeneity [illegible]	[illegible]	HR	Heterogeneity I^2	Heterogeneity H
Design Heterogeneity	Landy et al. (2021)[77]	Extreme Offers Reduce Trust (Replication)	$Q(12) = 366.3$ $p < 0.001$	☑	$\tau = 0.507$ $CI_{95\%}$ [0.358, 0.847]	0.088	5.763	$I^2 = 97.1$ $CI_{95\%}$ [94.3, 98.9]	$H = 5.849$ $CI_{95\%}$ [4.192, 9.687]
Design Heterogeneity	Landy et al. (2021)[77]	Moral Praise for Needless Work	$Q(12) = 157.7$ $p < 0.001$	☑	$\tau = 0.297$ $CI_{95\%}$ [0.205, 0.506]	0.074	4.005	$I^2 = 94.1$ $CI_{95\%}$ [88.5, 97.9]	$H = 4.128$ $CI_{95\%}$ [2.946, 6.912]
Design Heterogeneity	Landy et al. (2021)[77]	Moral Praise for Needless Work (Replication)	$Q(12) = 128.0$ $p < 0.001$	☑	$\tau = 0.234$ $CI_{95\%}$ [0.159, 0.402]	0.073	3.207	$I^2 = 91.1$ $CI_{95\%}$ [82.6, 96.8]	$H = 3.359$ $CI_{95\%}$ [2.395, 5.596]
Design Heterogeneity	Landy et al. (2021)[77]	Proximal Authorities Drive Legitimacy	$Q(11) = 87.2$ $p < 0.001$	☑	$\tau = 0.206$ $CI_{95\%}$ [0.135, 0.366]	0.077	2.682	$I^2 = 87.8$ $CI_{95\%}$ [75.5, 95.8]	$H = 2.863$ $CI_{95\%}$ [2.022, 4.886]
Design Heterogeneity	Landy et al. (2021)[77]	Proximal Authorities Drive Legitimacy (Replication)	$Q(11) = 47.3$ $p < 0.001$	☑	$\tau = 0.140$ $CI_{95\%}$ [0.084, 0.264]	0.075	1.883	$I^2 = 78.0$ $CI_{95\%}$ [55.7, 92.6]	$H = 2.132$ $CI_{95\%}$ [1.503, 3.685]
Design Heterogeneity	Landy et al. (2021)[77]	Deontological Judgments Predict Happiness	$Q(12) = 47.6$ $p < 0.001$	☑	$\tau = 0.149$ $CI_{95\%}$ [0.089, 0.272]	0.086	1.741	$I^2 = 75.2$ $CI_{95\%}$ [51.7, 91.0]	$H = 2.008$ $CI_{95\%}$ [1.439, 3.335]
Design Heterogeneity	Landy et al. (2021)[77]	Deontological Judgments Predict Happiness (Rep.)	$Q(12) = 85.0$ $p < 0.001$	☑	$\tau = 0.212$ $CI_{95\%}$ [0.140, 0.369]	0.085	2.484	$I^2 = 86.1$ $CI_{95\%}$ [72.9, 94.9]	$H = 2.678$ $CI_{95\%}$ [1.920, 4.433]
Analytical Heterogeneity	Hoogeveen et al. (2022)[79]	Cultural Norms	$Q(100) = 414.7$ $p < 0.001$	☒	$\tau = 0.018$ $CI_{95\%}$ [0.016, 0.026]	0.008	2.343	$I^2 = 84.6$ $CI_{95\%}$ [81.6, 92.1]	$H = 2.547$ $CI_{95\%}$ [2.333, 3.557]
Analytical Heterogeneity	Hoogeveen et al. (2022)[79]	Well-Being	$Q(98) = 1586.7$ $p < 0.001$	☒	$\tau = 0.039$ $CI_{95\%}$ [0.035, 0.049]	0.010	3.957	$I^2 = 94.0$ $CI_{95\%}$ [92.8, 96.2]	$H = 4.081$ $CI_{95\%}$ [3.728, 5.108]
Analytical Heterogeneity	Huntington-Klein et al. (2021)[80]	Health Insurance	$Q(6) = 21.1$ $p = 0.002$	☒	$\tau = 0.019$ $CI_{95\%}$ [0.014, 0.124]	0.014	1.404	$I^2 = 66.4$ $CI_{95\%}$ [52.3, 98.8]	$H = 1.724$ $CI_{95\%}$ [1.447, 9.038]
Analytical Heterogeneity	Huntington-Klein et al. (2021)[80]	Compulsory Education	$Q(6) = 227.4$ $p < 0.001$	☒	$\tau = 0.015$ $CI_{95\%}$ [0.009, 0.034]	0.003	5.786	$I^2 = 97.1$ $CI_{95\%}$ [93.1, 99.4]	$H = 5.872$ $CI_{95\%}$ [3.814, 13.293]
Analytical Heterogeneity	Silberzahn et al. (2018)[81]	Skin Tone and Red Cards	$Q(28) = 14361.6$ $p < 0.001$	☒	$\tau = 0.128$ $CI_{95\%}$ [0.081, 0.168]	0.010	12.655	$I^2 = 99.4$ $CI_{95\%}$ [98.5, 99.6]	$H = 12.695$ $CI_{95\%}$ [8.088, 16.621]

Table S2. Alternative measures quantifying heterogeneity in multi-analyst studies. For each multi-analyst study in our review (i.e., studies pertaining to analytical heterogeneity), we report the standard deviation of effect sizes across analysts, $SD(y_i)$, as a proxy of between-study variation, and the mean of the standard errors of the effect size estimates, $M(se_i)$, as a proxy for the average within-study variation. The relative measure to quantify heterogeneity proposed by Huntington-Klein et al.[80], denoted as HR_P, is defined as $HR_P = SD(y_i) \div M(se_i)$. In addition, we report the "H-equivalent" based on HR_P, denoted as H_P, defined as $H_P = (1 + HR_P^2)^{0.5}$. To facilitate comparability, the table also reports estimates for the between-study variation (τ), the within-study variation (σ), the heterogeneity measure H, and the alternative heterogeneity measure $HR = \tau \div \sigma$ obtained from random-effects meta-analyses.

		Random-Effects Meta-Analysis				*Huntington-Klein et al.'s Proxies*			
Study/Paper	*Hypothesis*	τ	σ	*HR*	*H*	$SD(y_i)$	$M(se_i)$	HR_P	H_P
Hoogeveen et al. (2022)[79]	Cultural Norms	0.018	0.008	2.343	2.547	0.039	0.014	2.701	2.880
Hoogeveen et al. (2022)[79]	Well-Being	0.039	0.010	3.957	4.081	0.049	0.014	3.487	3.628
Huntington-Klein et al. (2021)[80]	Health Insurance	0.019	0.014	1.404	1.724	0.059	0.019	3.073	3.231
Huntington-Klein et al. (2021)[80]	Compulsory Education	0.015	0.003	5.786	5.872	0.015	0.004	3.984	4.107
Silberzahn et al. (2018)[81]	Skin Tone and Red Cards	0.128	0.010	12.655	12.695	0.256	0.173	1.478	1.785
